# A randomised controlled trial to assess the clinical effectiveness and safety of the endometrial scratch procedure prior to first-time IVF, with or without ICSI

**DOI:** 10.1093/humrep/deab041

**Published:** 2021-05-29

**Authors:** Mostafa Metwally, Robin Chatters, Munya Dimairo, Stephen Walters, Clare Pye, David White, Priya Bhide, Tim Chater, Ying Cheong, Meenakshi Choudhary, Tim Child, Andrew Drakeley, Isaac Evbuomwan, Tarek Gelbaya, Jan Grace, Philip Harris, Susan Laird, Sarah Martins da Silva, Lamiya Mohiyiddeen, Kirsty Pemberton, Nick Raine-Fenning, Madhurima Rajkhowa, Tracey Young, Judith Cohen

**Affiliations:** 1 Obstetrics, Gynaecology & Neonatology, Sheffield Teaching Hospitals NHS Foundation Trust and The University of Sheffield, Sheffield, S10 2JF, UK; 2 Sheffield Clinical Trials Research Unit, The University of Sheffield, Sheffield, S1 4DA, UK; 3 School of Health and Related Research (ScHARR), The University of Sheffield, Sheffield, S1 4DA, UK; 4 Fertility Centre, Homerton University Hospital NHS Foundation Trust, Clapton, E9 6SR, UK; 5 Faculty of Medicine, University of Southampton, Southampton, SO17 1BJ, UK; 6 Oxford Fertility, The Fertility Partnership, Nuffield Department of Women’s and Reproductive Health, University of Oxford, Oxford, OX4 2HW, UK; 7 Newcastle Fertility Centre at Life, The Newcastle Upon Tyne Hospitals NHS Foundation Trust, Newcastle, NE1 4EP, UK; 8 The Hewitt Fertility Centre, Liverpool Women’s NHS Foundation Trust, Liverpool, L8 7SS, UK; 9 Gateshead Fertility Unit, Gateshead Health NHS Foundation Trust, Gateshead, NE9 6SX, UK; 10 Leicester Fertility Centre, University Hospitals of Leicester NHS Trust, Leicester, LE1 5WW, UK; 11 Assisted Conception Unit, Guy's and St Thomas' NHS Foundation Trust, London, SE1 9RT, UK; 12 Fertility Fusion, Wrightington, Wigan & Leigh Teaching Hospitals NHS Foundation Trust, Appley Bridge, WB6 9EP, UK; 13 Faculty of Health and Wellbeing, Sheffield Hallam University, Sheffield, S1 1WB, UK; 14 Reproductive Medicine Research Group, University of Dundee, Dundee DD1 4HN, UK; 15 Faculty of Biology, Medicine and Health, The University of Manchester, Manchester, M13 9PL, UK; 16 School of Medicine, University of Nottingham, Nottingham, NG7 2UH, UK; 17 Care Birmingham, Care Fertility Ltd., Birmingham, B15 3DP, UK; 18 Hull Health Trials Unit, The University of Hull, Hull, HU6 7RX, UK

**Keywords:** endometrial scratch, assisted reproduction, IVF, live birth, randomised controlled trial

## Abstract

**STUDY QUESTION:**

What is the clinical-effectiveness and safety of the endometrial scratch (ES) procedure compared to no ES, prior to usual first time in vitro fertilisation (IVF) treatment?

**SUMMARY ANSWER:**

ES was safe but did not improve pregnancy outcomes when performed in the mid-luteal phase prior to the first IVF cycle, with or without intracytoplasmic sperm injection (ICSI).

**WHAT IS KNOWN ALREADY:**

ES is an ‘add-on’ treatment that is available to women undergoing a first cycle of IVF, with or without ICSI, despite a lack of evidence to support its use.

**STUDY DESIGN, SIZE, DURATION:**

This pragmatic, superiority, open-label, multi-centre, parallel-group randomised controlled trial involving 1048 women assessed the clinical effectiveness and safety of the ES procedure prior to first time IVF, with or without ICSI, between July 2016 and October 2019.

**PARTICIPANTS/MATERIALS, SETTING, METHODS:**

Participants aged 18–37 years undergoing their first cycle of IVF, with or without ICSI, were recruited from 16 UK fertility clinics and randomised (1:1) by a web-based system with restricted access rights that concealed allocation. Stratified block randomisation was used to allocate participants to TAU or ES in the mid-luteal phase followed by usual IVF with or without ICSI treatment. The primary outcome was live birth after completing 24 weeks gestation within 10.5 months of egg collection.

**MAIN RESULTS AND THE ROLE OF CHANCE:**

In total, 1048 women randomised to TAU (n = 525) and ES (n = 523) were available for intention to treat analysis. In the ES group, 453 (86.6%) received the ES procedure. IVF, with or without ICSI, was received in 494 (94.1%) and 497 (95.0%) of ES and TAU participants respectively. Live birth rate was 37.1% (195/525) in the TAU and 38.6% (202/523) in the ES: an unadjusted absolute difference of 1.5% (95% CI −4.4% to 7.4%, *P* = 0.621). There were no statistical differences in secondary outcomes. Adverse events were comparable across groups.

**LIMITATIONS, REASONS FOR CAUTION:**

A sham ES procedure was not undertaken in the control group, however, we do not believe this would have influenced the results as objective fertility outcomes were used.

**WIDER IMPLICATIONS OF THE FINDINGS:**

This is the largest trial that is adequately powered to assess the impact of ES on women undergoing their first cycle of IVF. ES was safe, but did not significantly improve pregnancy outcomes when performed in the mid-luteal phase prior to the first IVF or ICSI cycle. We recommend that ES is not undertaken in this population.

**STUDY FUNDING/COMPETING INTEREST(S):**

Funded by the National Institute of Health Research. Stephen Walters is an National Institute for Health Research (NIHR) Senior Investigator (2018 to present) and was a member of the following during the project: National Institute for Health Research (NIHR) Health Technology Assessment (HTA) Clinical Trials and Evaluation Committee (2011–2017), NIHR HTA Commissioning Strategy Group (2012 to 2017); NIHR Programme Grants for Applied Research Committee (2020 to present); NIHR Pre doctoral Fellowship Committee (2019 to present). Dr. Martins da Silva reports grants from AstraZeneca, during the conduct of the study; and is Associate editor of Human Reproduction and Editorial Board member of Reproduction and Fertility. Dr. Bhide reports grants from Bart's Charity and grants and non-financial support from Pharmasure Pharmaceuticals outside the submitted work.

**TRIAL REGISTRATION NUMBER:**

ISRCTN number: ISRCTN23800982.

**TRIAL REGISTRATION DATE:**

31 May 2016

**DATE OF FIRST PATIENT’S ENROLMENT:**

04 July 2016

## Introduction

IVF and ICSI are widely used assisted reproductive technologies (ARTs) for women who are unable to conceive naturally. Success rates are modest, with an overall live birth rate (LBR) of 27% in the UK, with some evidence to suggest that worldwide success rates have been decreasing over recent years (Gleicher *et al.*, 2019). In an attempt to increase their chances of success, couples undergoing IVF or ICSI can select from a bewildering choice of ‘add-ons’, most of which lack evidence to support their benefits (Macklon *et al.*, 2019; Wise, 2019). One such add-on is the endometrial scratch (ES) procedure, which involves endometrial biopsy (‘scratching’) with a pipelle or similar sampling device. Because ES was first recognised as a potential intervention to increase the chance of implantation (Barash *et al.*, 2003), several biological hypotheses for a beneficial effect caused by inducing mechanical endometrial trauma have been suggested such as the release of inflammatory mediators, modulation of endometrial genes involved in membrane stability and enhancement of endometrial angiogenesis (Kalma *et al.*, 2009; Gnainsky *et al.*, 2010; Yang *et al.*, 2019).

Previous studies have examined the role of ES in various infertile populations but have focussed mainly on the recurrent implantation population (Raziel *et al.*, 2007; Huang *et al.*, 2011; El-Toukhy *et al.*, 2012). There have not been any high-quality randomised controlled trials (RCTs) adequately powered to assess the effectiveness of ES in women undergoing their first cycle of IVF/ICSI despite the use of the procedure in this particular population in clinical practice. Several controlled trials have identified contradictory evidence, with a 2019 systematic review by Vitagliano *et al.* (2019) identifying insufficient evidence to support the benefits of ES, concluding that an effect could not be ruled out. Many of these previous studies included heterogeneous patients, including both women undergoing their first IVF cycle, and women undergoing subsequent cycles, and were therefore not powered to detect clinically worthwhile effects in those undergoing their first IVF cycle (Nastri *et al.*, 2013; Yeung *et al.*, 2014; Lensen *et al.*, 2019; Mackens *et al.*, 2020). The two largest trials to focus specifically on women undergoing their first cycle of IVF included 418 and 300 participants and found significant increases in IVF success in women that received ES (Mahran *et al.*, 2016; Maged *et al.*, 2018) but, according to the Vitagliano *et al.* review, the risk of bias was deemed to be high in one study (Mahran *et al.*, 2016), and the other did not follow-up participants until delivery (Maged *et al.*, 2018; Vitagliano *et al.*, 2019). A high-quality RCT is required to definitively conclude if the ES procedure is effective and safe (Vitagliano *et al.*, 2019).

Despite the lack of evidence, ES is still provided to some women undergoing their first cycle of IVF with or without ICSI in fertility centres internationally (Lensen *et al.*, 2016). This definitive RCT assessed the clinical effectiveness and safety of the ES procedure compared to treatment as usual (TAU) in women undergoing their first IVF cycle, with or without ICSI.

## Materials and methods

### Trial design and oversight

We conducted a randomised, two-arm, superiority, open-label, parallel-group multicentre clinical trial, at 16 fertility units in the UK, two of which were run privately. The trial was designed to be pragmatic on the basis of PRECIS-2 criteria (Supplementary Table SI; Loudon *et al.*, 2015). The original trial protocol has been published previously (Pye *et al.*, 2018) and the latest protocol with amendments is accessible (Metwally *et al.*, 2020). A list of protocol amendments, with reasons, can be found in Supplementary Table SII.

Independent Trial Steering and Data Monitoring and Ethics Committees (TSC/DMEC) provided trial oversight. The trial had no interim analyses but the DMEC reviewed unblinded safety and outcome data every 6 months. A 6-month internal pilot assessed the feasibility of the trial. The trial was funded by the National Institute for Health Research (NIHR HTA 14/08/45), who had no role in the design, conduct, analysis or reporting of the trial. Ethics approval was granted by South Central Berkshire Research Ethics Committee (16/SC/0151) and the trial was registered with ISRCTN (ISRCTN23800982). The authors assume responsibility for the accuracy and completeness of the data and analyses, as well as adherence to the protocol and interpretation of results.

### Participants

Participants were women aged 18–37 years (inclusive); were undergoing their first cycle of IVF, with or without ICSI; were expected to be using fresh embryos and a single embryo transfer (SET); had a regular ovulatory menstrual cycle defined by clinical judgement or with ovulatory levels of midluteal serum progesterone, normal uterine cavity assessed by transvaginal sonography at screening, with no endometrial abnormalities that would require treatment to facilitate pregnancy (such as suspected intrauterine adhesions, uterine septae, submucosal fibroids or intramural fibroids exceeding 4 cm in diameter), good ovarian reserve assessed clinically, biochemically (FSH < 10 UI/L) and normal follicular phase oestradiol levels and/or normal anti-Müllerian hormone levels or sonographically (antral follicle count) and no history of previous radiotherapy or chemotherapy; had no relevant vaginal/uterine infections; and, if randomised to receive ES, were willing to use a barrier method of contraception prior to the procedure if necessary. Participants were excluded if they had received previous trauma to the endometrium (resection of uterine septum, intrauterine adhesions, or recent resection of significant submucous fibroids), had a BMI of 35 Kg/m^2^ or greater, were participating in another interventional fertility study, or had grade 4 endometriosis. All laboratory or ultrasound standards were based on local reference ranges. From July 2017, the eligibility criteria were altered so that participants undergoing ultra-long protocols were excluded, the use of which is commonly associated with severe endometriosis which may have an adverse effect on implantation and hence may have a confounding effect on the results. Those having other endometrial procedures (e.g. endometrial biopsy for the collection of natural killer cells) were also excluded.

### Trial procedures

Following informed consent, baseline assessments were undertaken prior to randomisation. Individuals were informed that participation in the trial may delay the start of their IVF cycle, if randomisation was being undertaken close to the start of their treatment. IVF was only delayed where necessary, with the agreement of both the patient and fertility team, in order to allow the ES to be scheduled prior to IVF.

At a timepoint between the participant’s initial IVF clinic consultation and the start of their IVF cycle, participants were randomly assigned (1:1) to the intervention or control arms by a doctor or nurse at the fertility unit using a web-based randomisation system with restricted access rights that concealed allocation. The trial statistician generated the randomisation sequence using a computer via a web-based system, but the access rights of the randomisation system did not allow this individual, or the research staff, to access the generated randomisation sequence. Stratified block randomisation was used, with randomly permuted blocks of sizes 2, 4, and 6 stratified by site and planned IVF/ICSI (antagonist or long). Block sizes were masked to the research team except for the trial statistician who was not involved in the screening and randomisation process. The trial statisticians and health economist were blinded and did not have access to patient level data during the trial, but blinding of site staff or participants was not possible owing to the nature of the intervention. Another statistician responsible for preparing unblinded summaries for the DMEC had no role in the day-to-day conduct of the trial.

Those allocated to the intervention arm received the ES procedure, which was undertaken in the mid-luteal phase (defined as 5–7 days before the expected next period, or 7–9 days after a positive ovulation test) of the menstrual cycle preceding IVF/ICSI by a suitably qualified doctor or nurse. Participants were required to use a barrier method of contraception (if necessary) in the menstrual cycle in which the ES was performed. ES was performed by inserting a speculum into the vagina and the cervix was exposed and cleaned. A pipelle sampler or similar device was then inserted into the cavity of the uterus and negative pressure was applied by withdrawal of the plunger. The sampler was then rotated and withdrawn 3 to 4 times so that tissue appeared in the transparent tube. The sampler and speculum were then removed. Pain ratings and tolerability were then collected from the patient.

Participants randomised to the control arm (TAU) received IVF treatment, with or without ICSI, in line with the usual care practice of their fertility unit. Those randomised to the ES arm received usual IVF treatment, with or without ICSI, in the menstrual cycle after the ES procedure.

Participants were followed up via telephone by a member of the research team based at the fertility unit for up to 10.5 months post egg collection. This time frame was selected to allow for any resulting pregnancy to reach full-term to observe a live birth outcome and 6 weeks postpartum follow-up of babies born. Egg collection was used as the starting point of the follow-up time frame rather than randomisation to standardise follow-up across treatment groups, as participants in the ES were expected to have a delay in the start of their IVF cycle after randomisation to allow ES to take place. Participants were not followed up if they were discontinued from the trial (pregnancy not achieved following first embryo transfer, end of pregnancy, investigator decision), withdrew or were lost to follow-up (Supplementary Fig. S4).). For all participants, details of the IVF/ICSI treatments received and their initial outcome (e.g. the number of eggs collected, quality of embryos transferred, implantation, and pregnancy) were collected from the patient’s medical records. Following a positive pregnancy test, follow-up was undertaken via telephone at 3 and 6 months post egg collection and 6 weeks post-partum to collect pregnancy-related outcomes and adverse events (AEs). Participants who became pregnant naturally prior to their first embryo transfer were followed up for the duration of their pregnancy. In case of cycles where all embryos had been frozen, or where the start of IVF had been delayed, the outcome of the first embryo transfer was collected if it had occurred within the overall study data collection period: The outcomes of such pregnancies were collected from the participant’s medical notes in order to minimise missing data on the primary outcome.

### Outcomes

The primary outcome was live birth (defined as live birth after completion of 24 weeks gestation). The secondary outcomes were implantation (based on a positive serum beta-hCG on approximately day 14 following egg collection, or by a positive urine pregnancy test), clinical pregnancy (an observation of viable intrauterine pregnancy with a positive heart pulsation seen on ultrasound at/after 8 weeks gestation), miscarriage (as measured by spontaneous pregnancy loss, including pregnancy of unknown location prior to 24 weeks gestation, within the 10.5 month post egg collection follow-up period), ectopic pregnancy (as measured by the rate of pregnancy outside the normal uterine cavity), multiple birth (defined as the birth of more than one living foetus after completed 24 weeks gestation), preterm delivery (as measured by live birth after 24 weeks and before 37 weeks gestation within the 10.5 month post egg collection follow-up period), stillbirth (based on the delivery of a stillborn foetus showing no signs of life after 24 weeks gestation within the 10.5 month post egg collection follow-up period), and details of the participant’s IVF cycle, including number of eggs retrieved and number of embryos generated. The quality of embryos transferred was assessed by an embryologist using grading systems as presented in Supplementary Tables SIII and SIV.

For those participants who received the ES, tolerability of the procedure (yes/no) and post-procedure pain (using a Likert scale between 0 and 10 collected within 30 minutes of the ES, and at 1 day and 7 days post ES) were recorded.

Safety outcomes were collected for the duration of participation in the trial and included any untoward medical event in the mother and, in the baby, neonatal death or a severe congenital abnormality [defined as an abnormality not listed on the European Surveillance of Congenital Anomalies (EUROCAT) minor anomalies list (EUROCAT, 2013)] up to 6 weeks post-partum. Expectedness was assessed for all events, with a list of expected events pre-specified in the protocol as either expected AEs, or expected serious adverse events (SAEs). Expected events were those that were considered to be expected during pregnancy or an IVF cycle (e.g. expected AEs included nausea, hot flushes; expected SAEs included hospitalisation for rest or gestational diabetes). Any event reported that was not on the list of expected events was therefore classed as an unexpected event. From January 2017, the reporting of AEs was altered, in that events in the mother related to the birth of a baby or the process of birth were no longer classed as AEs or reported within the trial.

### Statistical analysis

LBR was defined as the proportion of women achieving at least one live birth, with the number of women randomised as the denominator. A 30% control LBR was assumed based on data from the Human Fertilisation and Embryology Authority (HFEA). A 10% absolute increase in LBR was viewed to be of sufficient clinical importance to change practice. With continuity correction, the study required a total of 1044 participants (522 per group) to preserve a 90% power to detect a 10% absolute difference for a 5% two-sided test while accounting for a 5% dropout rate. This inflated sample size accounted for uncertainty around the assumed control LBR.

All statistical methods are detailed in a prespecified Statistical Analysis Plan (Dimairo *et al.*, 2020). In sum, the primary analysis was based on intention to treat (ITT) analysis population. For the primary outcome (live birth), the treatment effect of interest was the absolute difference in LBRs between groups. Participants with missing data on live birth were assumed to have not achieved a live birth (worst-case scenario). Normal approximation to the binomial distribution was used to calculate the 95% CI around the differences in LBRs. The associated *p*-value was calculated using Pearson’s chi-squared test. Corresponding unadjusted odds ratio (OR) and unadjusted relative risk with 95% CIs were estimated using simple logistic regression and Binomial generalized linear model with a log link function, respectively.

Sensitivity analysis was performed by adjusting for fixed stratification factors (site and planned treatment protocol) and potential prognostic factors (history of pregnancy, yes or no; age; BMI; duration of infertility; smoking status, yes or no). The adjusted OR (aOR) with 95% CI were estimated using a multiple logistic regression model and adjusted relative risk (aRR) with 95% CI using a Binomial generalized linear model with a log link function. The adjusted absolute difference (aAD) in LBRs with 95% CI were postestimated via margins using the delta method (Norton *et al.*, 2013) after fitting a Binomial generalized linear model with a log link function.

Further sensitivity analysis on live birth was performed assuming the best-case scenario (that is, those with missing live birth data but known to be pregnant were assumed to have achieved a live birth) and using the complete case population (only those with a known live birth outcome).

Potential heterogeneity in the effect of ES on the primary outcome assuming the worst-case scenario was explored through prespecified subgroup analyses via interaction tests and forest plots.

Prespecified subgroups were: day of embryo transfer (day 2, 3, 4, 5 or 6), fertilisation method (IVF, ICSI or IVF and ICSI split), treatment protocol (long or antagonist), embryo transfer (single or double), nature of embryo transferred (fresh or frozen), previous history of miscarriages (0–2 or ≥3), and cycle programming (yes or no). The aOR with associated 95% CI and interaction p-value were estimated using a multiple logistic regression model that included an interaction term between the treatment group and subgroup as well as fixed stratification factors and potential prognostic factors stated above. However, for subgroups relating to the treatment protocol and history of miscarriage, the planned treatment protocol and history of pregnancy covariates were excluded in the model, respectively. Because of small numbers of events and sample sizes within subgroups, the aAD with 95% CIs were not estimated as the postestimation of margins using the delta method failed to converge.

The per protocol (PP) analysis population included women who met all inclusion criteria as stipulated in the protocol; received the allocated treatment; did not achieve a pregnancy before treatment; completed the fertility treatment cycle and successfully generated embryos; used contraception before the ES procedure; had a fresh embryo transfer, and were treated only using the antagonist or long protocols. PP analysis on the primary outcome was performed assuming a worst-case scenario using methods described for the primary ITT analysis.

Unadjusted analyses on all secondary outcomes were carried out in the same manner as described for the primary outcome. These results were not adjusted for multiple testing and all hypothesis tests were performed at 5% two-sided significance level.

Analysis of safety outcomes in women and born babies was based on randomised participants with informed consent and treatment as received rather than allocated. Unexpected AEs and SAEs that occurred after receiving the treatment were summarised per group using the numbers and proportions of women who reported at least one event as well as the total number of repeated events. The numbers of repeated events per woman were analysed using a Negative Binomial regression model accounting for follow-up period to estimate the incidence rate (IR) per treatment group and IR ratio (IRR) with 95% CI. Sensitivity analysis was performed by including all unexpected AEs and SAEs reported at any point during the trial after randomisation. Events that occurred between receiving ES and IVF in the ES group only were summarised descriptively. Finally, in women who have a positive pregnancy test, safety outcomes recorded in born babies were summarised using the numbers and proportions per group. The unadjusted absolute differences (uADs) in proportions between groups were calculated and 95% CIs obtained using the Normal approximation to the Binomial distribution.

All analyses were performed in Stata version 16.1 (StataCorp. 2019. *Stata Statistical Software: Release 16*. College Station, TX: StataCorp LLC).

## Results

### Participants

**Figure 1.**
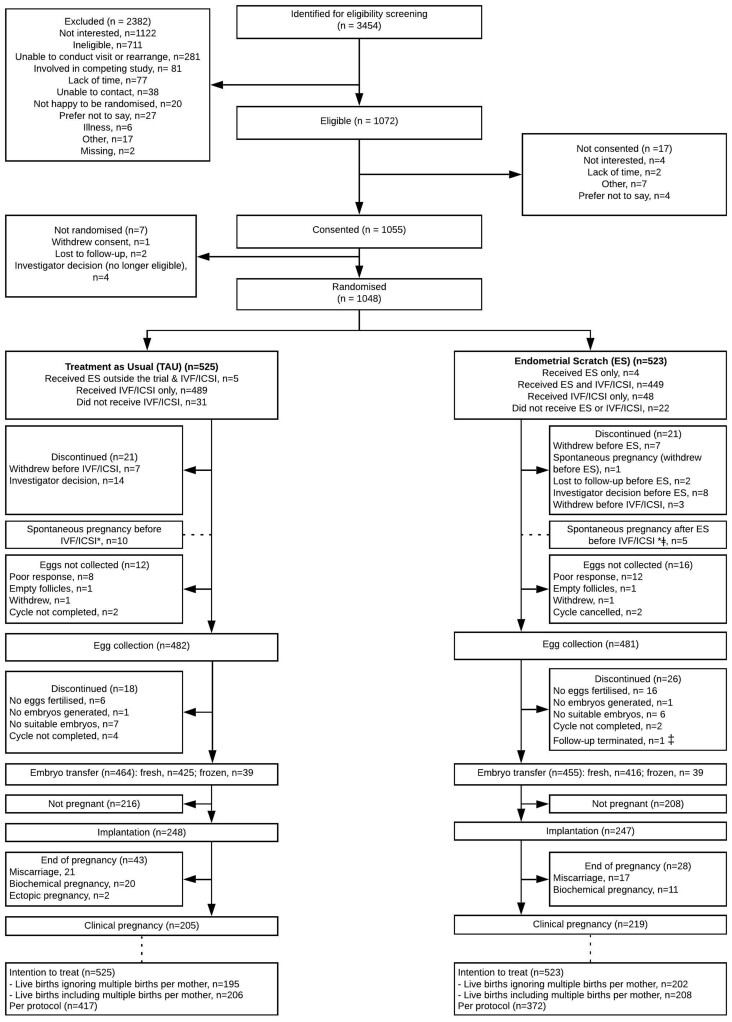
**Eligibility screening and follow-up of study participants.** ‡ one spontaneous pregnancy result in ectopic pregnancy; * note the spontaneous pregnancies were followed-up; ‡ follow-up was terminated for one participant that did not commence IVF following randomisation—follow-up was therefore terminated at the end of the follow-up phase of the trial.

Participants were recruited between 4th July 2016 and 24th October 2018, with follow-up continuing until 24th October 2019. A total of 3454 women were identified for screening; of which 1048 (30.3%) were randomised to either TAU (n = 525) or ES (n = 523) (Fig. 1). The characteristics of randomised participants were very similar between groups (Table I).

**Table I:** Baseline demographics and characteristics of randomised participants.

Demographic or characteristic	TAU	ES	Total
	(n = 525)	(n = 523)	(n = 1048)
Fertility unit, n (%)			
Birmingham	32 (6.1%)	31 (5.9%)	63 (6.0%)
Dundee	25 (4.8%)	26 (5.0%)	51 (4.9%)
Gateshead	31 (5.9%)	30 (5.7%)	61 (5.8%)
Guys & St Thomas	28 (5.3%)	27 (5.2%)	55 (5.2%)
Homerton	14 (2.7%)	15 (2.9%)	29 (2.8%)
Leeds	61 (11.6%)	61 (11.7%)	122 (11.6%)
Leicester	25 (4.8%)	26 (5.0%)	51 (4.9%)
Liverpool	54 (10.3%)	56 (10.7%)	110 (10.5%)
Manchester	62 (11.8%)	63 (12.0%)	125 (11.9%)
Newcastle	28 (5.3%)	28 (5.4%)	56 (5.3%)
Nottingham	3 (0.6%)	3 (0.6%)	6 (0.6%)
Oxford	27 (5.1%)	26 (5.0%)	53 (5.1%)
Sheffield	78 (14.9%)	75 (14.3%)	153 (14.6%)
South Tees	15 (2.9%)	15 (2.9%)	30 (2.9%)
Southampton	28 (5.3%)	29 (5.5%)	57 (5.4%)
Wrightington	14 (2.7%)	12 (2.3%)	26 (2.5%)
BMI (kg/m²)	(n = 525)	(n = 523)	(n = 1048)
Mean(SD)	24.5 (3.4)	24.5 (3.3)	24.5 (3.3)
Min, max	17.3, 35.0	16.8, 34.9	16.8, 35.0
Expected age at egg collection (years)	(n = 525)	(n = 523)	(n = 1048)
Mean(SD)	32.4 (3.4)	32.6 (3.4)	32.5 (3.4)
Min, max	21.5, 38.0	21.4, 38.1	21.4, 38.1
Actual age at egg collection (years) ‡	(n = 482)	(n = 481)	(n = 963)
Mean(SD)	32.4 (3.4)	32.7 (3.3)	32.5 (3.4)
Min, max	21.4, 38.8	21.4, 38.1	21.4, 38.8
Ethnicity, n (%)			
White (a)	472 (89.9%)	460 (88.0%)	932 (88.9%)
Asian/Asian British (b)	31 (5.9%)	47 (9.0%)	78 (7.4%)
Mixed/multiple ethnic groups (c)	7 (1.3%)	9 (1.7%)	16 (1.5%)
Black/African/Caribbean/Black British (d)	7 (1.3%)	3 (0.6%)	10 (1.0%)
Other ethnic group (e)	5 (1.0%)	4 (0.8%)	9 (0.9%)
Prefer not to say	2 (0.4%)	0 (0.0%)	2 (0.2%)
Unknown	1 (0.2%)	0 (0.0%)	1 (0.1%)
Current cigarettes smoker (f), n(%)	13 (2.5%)	11 (2.1%)	24 (2.3%)
Number of cigarettes per day	(n = 13)	(n = 10)	(n = 23)
Mean(SD)	6.2 (5.5)	8.2 (5.6)	7.1 (5.5)
Median(IQR)	3.0 (2.0, 10.0)	10.0 (2.0, 10.0)	7.0 (2.0, 10.0)
Min, max	1.0, 17.0	1.0, 20.0	1.0, 20.0
Alcohol drinker, n(%)	286 (54.5%)	278 (53.2%)	564 (53.8%)
Alcohol intake (units per week) ‡	(n = 279)	(n = 274)	(n = 553)
Mean(SD)	4.4 (3.2)	4.6 (4.1)	4.5 (3.7)
Median(IQR)	4.0 (2.0, 6.0)	3.0 (2.0, 6.0)	4.0 (2.0, 6.0)
Min, max	1.0, 18.0	1.0, 20.0	1.0, 20.0
Current recreational drug user, n(%)	0 (0.0%)	0 (0.0%)	0 (0.0%)
History of fertility treatment (not IVF), n(%)	89 (17.0%)	109 (20.8%)	198 (18.9%)
Fertility treatment received, n(%)	(n = 89)	(n = 109)	(n = 198)
IUI	55 (61.8%)	66 (60.6%)	121 (61.1%)
Clomid	31 (34.8%)	40 (36.7%)	71 (35.9%)
IUI and Clomid	0 (0.0%)	2 (1.8%)	2 (1.0%)
Donor insemination	1 (1.1%)	1 (0.9%)	2 (1.0%)
Tamoxifen and dostinex	1 (1.1%)	0 (0.0%)	1 (0.5%)
Had other significant medical conditions, n(%)	139 (26.5%)	122 (23.3%)	261 (24.9%)
History of any previous pregnancies	150 (28.6%)	155 (29.6%)	305 (29.1%)
Planned method of fertilisation, n(%)			
IVF	319 (60.8%)	316 (60.4%)	635 (60.6%)
ICSI	206 (39.2%)	207 (39.6%)	413 (39.4%)
Planned treatment protocol, n(%)			
Antagonist	313 (59.6%)	308 (58.9%)	621 (59.3%)
Long protocol	212 (40.4%)	215 (41.1%)	427 (40.7%)
Planned cycle programming, n(%)	131/313 (41.9%)	126/308 (40.9%)	257/621 (41.4%)
Cycle programming details	(n = 131)	(n = 126)	(n = 257)
Oral contraception	70 (53.4%)	67 (53.2%)	137 (53.3%)
Progestogens	54 (41.2%)	52 (41.3%)	106 (41.2%)
Oral oestrogen	7 (5.3%)	7 (5.6%)	14 (5.4%)
Duration of infertility (years) ‡‡	(n = 525)	(n = 523)	(n = 1048)
Mean(SD)	3.1 (1.7)	3.1 (1.9)	3.1 (1.8)
Median(IQR)	2.8 (2.0, 3.7)	2.8 (2.0, 3.5)	2.8 (2.0, 3.5)
Min, max	0.0, 15.0	0.0, 18.0	0.0, 18.0
Previous pregnancies (g), n(%)			
0	375 (71.4%)	368 (70.4%)	743 (70.9%)
1	103 (19.6%)	109 (20.8%)	212 (20.2%)
2	34 (6.5%)	33 (6.3%)	67 (6.4%)
3	10 (1.9%)	4 (0.8%)	14 (1.3%)
4	2 (0.4%)	5 (1.0%)	7 (0.7%)
≥5	1 (0.2%)	4 (0.8%)	5 (0.5%)
Previous miscarriages, n(%)			
0	442 (84.2%)	437 (83.6%)	879 (83.9%)
1	65 (12.4%)	68 (13.0%)	133 (12.7%)
2	12 (2.3%)	12 (2.3%)	24 (2.3%)
≥3	6 (1.1%)	6 (1.1%)	12 (1.1%)
Previous terminations, n(%)			
0	479 (91.2%)	471 (90.1%)	950 (90.6%)
1	41 (7.8%)	48 (9.2%)	89 (8.5%)
≥2	5 (1.0%)	4 (0.8%)	9 (0.9%)
Previous stillbirths, n(%)			
0	522 (99.4%)	520 (99.4%)	1042 (99.4%)
1	3 (0.6%)	3 (0.6%)	6 (0.6%)
Previous live births, n(%)			
0	501 (95.4%)	497 (95.0%)	998 (95.2%)
1	21 (4.0%)	22 (4.2%)	43 (4.1%)
≥2	3 (0.6%)	4 (0.8%)	7 (0.7%)
Previous ectopic pregnancies, n(%)			
0	508 (96.8%)	504 (96.4%)	1012 (96.6%)
1	13 (2.5%)	13 (2.5%)	26 (2.5%)
≥2	4 (0.8%)	6 (1.1%)	10 (1.0%)
Parity, n(%) †			
0	501 (95.4%)	498 (95.2%)	999 (95.3%)
1	21 (4.0%)	22 (4.2%)	43 (4.1%)
≥2	3 (0.6%)	3 (0.6%)	6 (0.6%)

TAU, treatment as usual; ES, endometrial scratch. ‡ Only in women with successful egg collection. Ethnicity classification: (a) English/Welsh/Scottish/Northern Irish/British, Irish, Gypsy or Irish Traveller, and any other White background; (b) Indian, Pakistani, Bangladeshi, Chinese, and any other Asian background; (c) White and Black Caribbean, White and Black African, White and Asian, and any other mixed/multiple ethnic groups background; (d) African, Caribbean, and any other Black/African/Caribbean/Black British background; (e) Arab, and any other ethnic group. ‡ A few alcohol drinkers had missing alcohol intake data. ‡‡ 12 had zero duration of infertility (these were women seeking treatment or couples in same-sex relationships without any known fertility problems). Parity is defined as the number of times a woman gave birth to a foetus (either a live or stillbirth) with a gestational age of at least 24 weeks; † parity could not be ascertained in eight births or stillbirths without gestational age: TAU (n = 3) and ES (n = 5). Preterm delivery is defined as a live birth after 24 weeks but before 37 gestational age (≥24 and <37). Current smoker relates to smoking cigarettes and not vaping.

### Uptake of interventions and acceptability of the ES procedure

In the ES group, 86.6% (453/523) received the ES procedure as PP and IVF/ICSI was administered to 497 (449 + 48) 95.0% who were randomised to ES (Supplementary Fig. S1). The median (interquartile range: IQR) time from ES to embryo transfer was 34.0 days (26.0, 42.0) ranging from 16.0 to 346.0 (outlier) (Supplementary Fig. S2). In total, 99.8% (448/449) viewed the ES procedure as tolerable (95% CI; 98.7%, 100.0%). The median (IQR) of pain rating scores within 30 minutes of the ES procedure, at 24 hours and day 7 after the ES procedure was 4.0 (2.0, 6.0), 1.0 (0.0, 3.0) and 0.0 (0.0, 0.0), respectively (Supplementary Fig. S3 and Supplementary Table SV).

In the TAU group, 94.1% (494/525) received IVF/ICSI. Only 1.0% (5/494) of IVF/ICSI recipients received the ES procedure outside the trial, indicating negligible contamination. Figure 1 and Supplementary Fig. S1 detail the reasons for not receiving allocated treatments.

### Treatment cycle characteristics

The treatment cycle characteristics of women who received IVF/ICSI were generally similar between TAU and ES groups before egg collection, at egg collection and embryo transfer (Supplementary Tables SVI, SVII and SVIII).

Response to IVF/ICSI treatment was high and similar between groups, with regards to the number of eggs collected, quality of the embryos that developed, and the proportion of patients undergoing SET (Supplementary Tables SVII and SVIII).

### Primary outcome

There was no significant difference in LBR between the groups. The LBR was 37.1% (195/525) in the TAU and 38.6% (202/523) in the ES groups, which gives an uAD of 1.5% (95% CI: –4.4% to 7.4%, *P* = 0.621). This unadjusted treatment effect was consistent with sensitivity analyses and very similar to adjusted results (Table II). The results of the PP analyses also demonstrated a non-significant effect and were similar across considered scenarios (Supplementary Table SIX). As such, the observed effect was not statistically significant and extremely unlikely to be of clinical importance.

**Table II:** The effect of ES on achieving a live birth (primary outcome).

Primary outcome	TAU n/N (%)	ES n/N (%)	Unadjusted treatment effect (95% CI)			*P*-value
			Absolute difference	Odds ratio	Relative risk	
LBR (worst-case)	195/525 (37.1%)	202/523 (38.6%)	1.5% (−4.4%, 7.4%)	1.06 (0.83, 1.37)	1.04 (0.89, 1.21)	0.621
Sensitivity analysis						
LBR (best-case)	197/525 (37.5%)	205/523 (39.2%)	1.7% (−4.2%, 7.6%)	1.07 (0.84, 1.38)	1.04 (0.90, 1.22)	0.578
LBR (complete case)	195/523 (37.3%)	202/520 (38.8%)	1.6% (−4.3%, 7.5%)	1.07 (0.83, 1.37)	1.04 (0.89, 1.22)	0.604

LBR, live birth rate; best-case scenario assumes that patients who were lost to follow-up or had unknown pregnancy outcome at the end of the trial for any reasons got pregnant and gave live birth. The complete case includes only those women with known pregnancy and a live birth outcome at the end of the trial.

**Figure 2.**
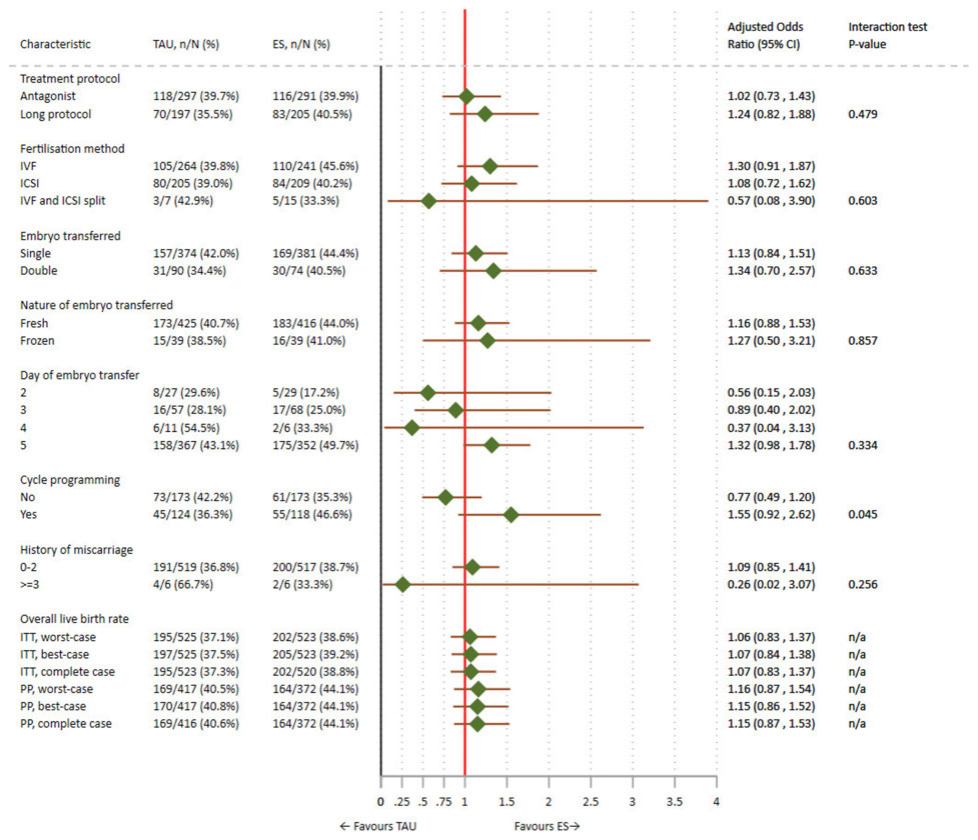
**The effect of ES on achieving live birth in prespecified subgroups**.

We did not find strong evidence to suggest heterogeneity in the effect of ES across prespecified subgroups (Fig. 2) although a potential for benefit could not be ruled out in women who had day 5 embryo transfers or those who underwent cycle programming with using oral contraception, progestogens, or oral oestrogen. However, these results were exploratory and readers should interpret them with extreme caution owing to the small numbers of participants and events within each subgroup.

### Secondary outcomes

**Figure 3.**
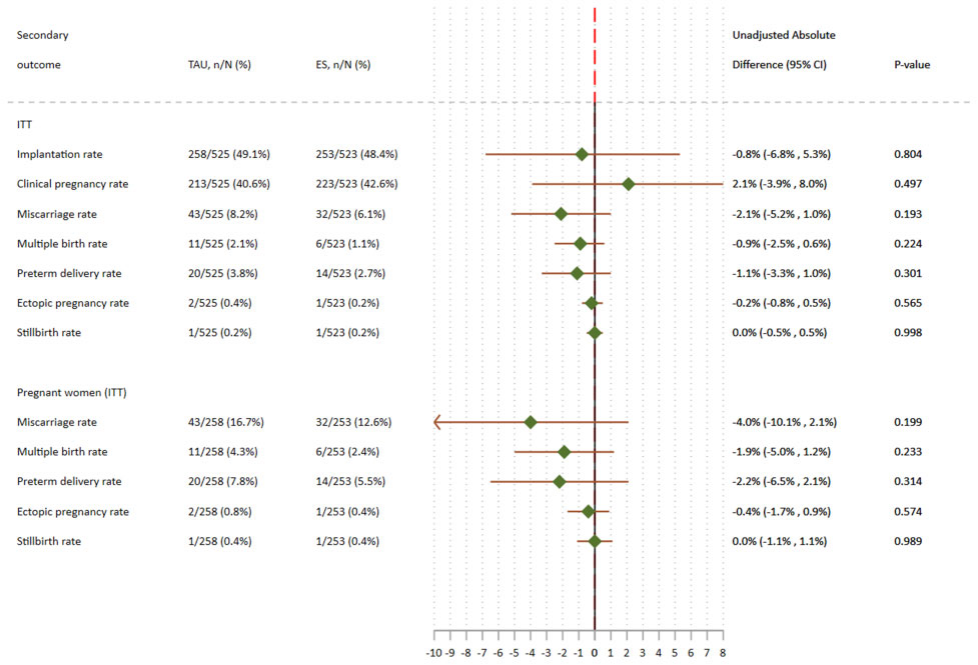
**The effect of ES on secondary outcomes.** Directional interpretation (favours TAU/favours ES) depends on the outcome.

There were no statistical differences in the rates of all secondary outcomes between groups (Fig. 3). The clinical pregnancy rate was 40.6% (213/525) in the TAU and 42.6% (223/523) in the ES, resulting in only a 2.1% uAD in favour of the ES procedure (95% CI: −3.9% to 8.0%; *P* = 0.497). Supplementary Tables SX and SXI present detailed results in ITT population and among pregnant women.

### Safety outcomes

Expected AEs (Supplementary Table SXII), unexpected AEs and SAEs (Supplementary Table SXIII) were similar between groups. Only 2.4% (11/458) women reported unexpected AEs that occurred after the delivery of ES but before receiving IVF/ICSI procedure (Supplementary Table SXIV).

Successful implantation was reported in 270 and 226 women who received TAU and ES, respectively. Three (1.1%) severe congenital abnormalities were reported in the TAU group only. No neonatal deaths were reported. SAEs in born babies were comparable between groups although we did find small reductions in low birthweight, very low birthweight and small for gestational age in the ES compared to the TAU group (Supplementary Table SXV).

## Discussion

In this multi-centre RCT involving women undergoing IVF/ICSI for the first time, the primary outcome was live birth for which the results from the primary and sensitivity analyses as well as subgroup analyses consistently failed to show evidence for benefit of ES. Results were also consistent regarding secondary outcomes.

The major strength of this study is the specific focus on one particular population to minimise heterogeneity. We excluded patients with potential factors that could influence endometrial quality (anovulation, BMI >35kg/m^2^ and severe endometriosis). External validity was maintained by recruiting participants from both National Health Service and private fertility units across the UK, including both long and antagonist protocols, and by allowing pragmatic delivery of ES. The study met its target sample size, with adequate power to detect a 10% difference between groups, which we considered to be sufficient to change clinical practice.

The potential confounding effect of embryo quality was addressed by including only good responders who were likely to have a SET. Indeed, our results show that most of participants responded well to stimulation and approximately 80% received a SET on day 5. However, 18% of participants still received a double embryo transfer (DET). The trial was conducted across 16 centres in the UK and therefore is a relatively accurate reflection of the national rate of SET versus DET during the study period and is consistent with recent HFEA data that report a 21% rate of DET across all cycles in those under 35 years of age in 2018 (HFEA, 2020). In our trial, the reasons for DET were identified as patient choice or the number and/or quality of the embryos not meeting the centres’ criteria for SET. Most participants, however, were indeed good responders who received a SET and therefore the effect of poor embryo quality and the replacement of more than one embryo when clinically indicated is unlikely to have introduced heterogeneity in our population.

The study did not include a sham procedure in the control group. However, we do not believe that this would have influenced our results as objective fertility outcomes such as those used in this study are unlikely to be influenced by a placebo effect. Only 1% of those allocated to the control arm received the scratch outside the study (i.e. the participant sought ES out owing to their desire to receive the procedure) indicating high compliance and negligible contamination of the control group.

Despite a lack of good evidence of benefit to support its use, some in the medical community have been quick to adopt ES, including for those undergoing their first IVF cycle (Lensen *et al.*, 2016). Several studies have been undertaken in ‘unselected’ populations and were not specifically powered towards the first-time IVF group (Nastri *et al.*, 2013; Yeung *et al.*, 2014; Eskew *et al.*, 2019; Lensen *et al.*, 2019); other studies have been undertaken in women undergoing their first IVF cycle but had relatively small sample sizes (Karimzade *et al.*, 2010; Yoldemir and Erenus, 2011; Liu *et al.*, 2017). A recent study by Lensen *et al.* (2019) included some participants undergoing their first IVF cycle, but was not powered specifically on LBR for the first IVF cycle population, and did not provide information on LBR in this particular group. Significant heterogeneity was introduced by including populations with different prognostic potential and a high proportion (26%) of frozen embryo transfers. Two of the largest RCTs to date in patients receiving first-time IVF (Mahran *et al.*, 2016; Maged *et al.*, 2018) included 418 and 300 participants respectively and identified significant increases in IVF success in women that received ES, but either did not follow-up participants until delivery or, according to a recent systematic review, were at a high risk of bias (Mahran *et al.*, 2016; Maged *et al.*, 2018; Vitagliano *et al.*, 2019).

We have identified two findings that require further clarification. First, we describe a decreased incidence of low birthweight, very low birthweight and small for gestational age in the ES group. To the best of our knowledge, this is the first time such an effect has been described, however, these results should be interpreted with extreme caution, owing to these events being highly correlated, the small numbers, and the lack of adjustment for other potential confounding factors. The results of a currently ongoing individual participant data analysis should be awaited before investigating further. Second, we identified two prespecified subgroups where the benefit of ES could not be ruled out (cycle programming and day 5 embryo transfer). However, the number of participants within these subgroups is too small to give reliable CIs and to allow conclusions to be draw regarding any potential positive effect.

In conclusion, this study provides evidence that, although safe and tolerable, ES does not significantly improve pregnancy outcomes and should not be offered to this group of women.

## Data availability

The data underlying this study will be shared on reasonable request to the corresponding author.

## Supplementary Material

deab041_Supplementary_Table_S1

deab041_Supplementary_Table_S2

deab041_Supplementary_Table_S3

deab041_Supplementary_Table_S4

deab041_Supplementary_Table_S5

deab041_Supplementary_Table_S6

deab041_Supplementary_Table_S7

deab041_Supplementary_Table_S8

deab041_Supplementary_Table_S9

deab041_Supplementary_Table_S10

deab041_Supplementary_Table_S11

deab041_Supplementary_Table_S12

deab041_Supplementary_Table_S13

deab041_Supplementary_Table_S14

deab041_Supplementary_Table_S15

deab041_Supplementary_Figure_S1

deab041_Supplementary_Figure_S2

deab041_Supplementary_Figure_S3

deab041_Supplementary_Figure_S4

## Notes

On behalf of the Endometrial Scratch Research Team.
