# Supplementary material for: A randomised controlled trial to assess the clinical effectiveness and safety of the endometrial scratch procedure prior to first-time IVF, with or without ICSI
Source: Hum Reprod. 2021 May 29;36(7):1841–53. doi: 10.1093/humrep/deab041 (PMC8213451; doi:10.1093/humrep/deab041)
Supplement: deab041_Supplementary_Table_S1 [file deab041_supplementary_table_s1.pdf]

Human Reproduction, pp. 1–1, 2021
doi:10.1093/humrep/deab041

human reproduction

SUPPLEMENTARY DATA

Supplementary Table SI PRECIS-2 tool as applied to a randomised controlled trial on the safety and benefit of endometrial scratch in a first IVF cycle.

	Domain	Score	Rationale
1	Eligibility Criteria	3	Patients only included if they are predicted to be good responders to treatment with single embryo transfer—differs from the patients who would receive the intervention in usual care.
2	Recruitment Path	5	Recruitment took place at routine care appointments.
3	Setting	5	Identical setting to usual care, including both National Health Service (NHS) and privately run fertility units.
4	Organisation intervention	4	A slight increase in resources needed to deliver the intervention in order to collect pain scores. However, no additional training was required, although sites referred to an Endometrial Scratch (ES) procedure standard operating procedure (SOP). The information sheets were provided to all participants regarding the ES procedure, which may not be provided during usual care.
5	Flex of experimental intervention –Delivery	4	There was flexible delivery in terms of timing (within limits) and exact protocol, but an SOP provided to ensure some consistency in the delivery of the ES intervention across centres.
6	Flex of experimental intervention –Adherence	5	There was no more than “usual” encouragement to undergo the intervention.
7	Follow-up	3	There were some follow-ups above usual care practice. The outcome of IVF cycles would usually be followed up by centres, but we undertook higher intensity follow-up than usual (e.g. 3 months, 6 months and 6 weeks post-partum follow-ups) and collected additional information (e.g. adverse events) more thoroughly and frequently than would be undertaken in usual care.
8	Outcome	5	Achieving pregnancy and a live birth is of obvious importance to participants.
9	Analysis	5	Intention to treat analysis used that included all randomised participants with informed consent regardless of circumstances after randomisation.
