# Supplementary material for: A randomised controlled trial to assess the clinical effectiveness and safety of the endometrial scratch procedure prior to first-time IVF, with or without ICSI
Source: Hum Reprod. 2021 May 29;36(7):1841–53. doi: 10.1093/humrep/deab041 (PMC8213451; doi:10.1093/humrep/deab041)
Supplement: deab041_Supplementary_Table_S2 [file deab041_supplementary_table_s2.pdf]

Human Reproduction, pp. 1–2, 2021
doi:10.1093/humrep/deab041

human reproduction

SUPPLEMENTARY DATA

Supplementary Table SII List of protocol amendments.

Amendment	Reason
**Substantial amendments**	
**Version 1 (25/02/2016) amended to version 1.1 (29/03/2016)**	
Clarification added that the outcome of the pregnancy will be confirmed with the patient's clinician before contacting the participant for follow-up information.	As requested by the ethics committee, in order to ensure participants are not contacted for follow-up who have had a negative pregnancy outcome.
**Version 1.1 (29/03/2016) amended to version 2 (09/05/2016)**	
The necessity to randomise participants no later than 1 month before the start of their IVF cycle (thus allowing randomisation at any point prior to IVF) was removed.	This time-frame was too restrictive and had a negative effect on recruitment.
Southampton was added as a site for the tissue substudy.	n/a
An inclusion criterion was added that women are expected to receive IVF/ICSI treatment using fresh embryos.	Those participants at entry into the trial that are expected to receive frozen embryo transfer are unlikely to be good responders to treatment, and are therefore ineligible for the trial.
The time point of the 9/10 month follow-up point was changed to 10.5 month (6 week post-partum).	The time point of the final follow-up was moved in order to allow neonatal deaths to be collected up to 6 weeks post partum.
Addition of a telephone follow-up pro-forma for Nurses to use when undertaking follow-up phone calls.	Incorporated in order to improve the replicability of the follow-ups, and also in order to improve the collection of source data from the follow-up phone calls.
Changes were made to the research sites listed (Newcastle, Guys, Nottingham, Oxford added, Bradford removed, change of PI in Sheffield and Leeds).	n/a
**Version 2 (09/05/2016) amended to version 3 (13/06/2016)**	
The eligibility criteria was altered—removal of cycle programming as an exclusion criterion.	Cycle programming is common practice in participating fertility units and exclusion of such participants was deemed unnecessary
Removal of stipulation that anti-inflammatory drugs cannot be taken prior to receiving the intervention.	General advise given to fertility patients as anti-inflammatory drugs should be avoided during treatment as they affect OHSS and renal function
Clarification that if a participant has not used a barrier method of contraception prior to the endometrial scratch, the IVF cycle should be delayed if the participant and fertility team agree to this, otherwise, the IVF should be undertaken as planned and the endometrial scratch not undertaken.	Added following queries from research sites.
Alteration to the subgroup analyses (correction to data collected for day of embryo transfer).	Error in previous versions of the protocol
Change of archiving time frame from 15 to 5 years.	Error in previous versions of the protocol
Unnecessary detail removed from SAE and AE section.	Improvement in wording of this section.
An AE and SAE collection time point added post procedure.	This was already being undertaken, but clarity was improved in the protocol.
**Version 3 (13/06/2016) amended to version 4 (04/10/2016)**	
Changes were made to the duration of the pilot study (from 6 months to 9 months) and changes to the stop/go criteria (removal of the necessity to set up at least 4 sites by the end of the pilot trial).	There were concerns at the time due to the new HRA approval process being implemented at the time. In agreement with the funder, the pilot study was altered to reflect the new challenges.
Detail added to follow-up process regarding process of following-up pain score text messages following no response from participant.	Added following identification that some participants were not responding to the initial text message.
Patient facing documents summarising both the Endometrial Scratch and E-Freeze trials were added.	Both trials were recruiting from a similar patient population at the same time. We therefore created joint patient facing documents in order to enable potential participants to make an informed choice regarding which trial to participate in.
Removal of requirement for HFEA consent to disclosure prior to informed consent into the trial.	This consent is not under the remit of the HFEA.
24 hour post intervention pain scale text renamed to "1 day".	To improve clarity.
**Version 4 (04/10/2016) amended to version 5 (20/07/2017)**	
	To improve clarity.

(continued)

Supplementary Table SII Continued

Amendment	Reason
The name of the 10.5 months follow-up was changed to "6 weeks post-partum".	
Clarification around the definition of a cycle of IVF within the trial (receiving any sort of stimulation).	To improve clarity, following queries from research sites as to when a participant is deemed to have started their IVF cycle.
Exclusion criteria added for participants undergoing protocols other than long or antagonist (i.e. ultra-long protocols)	Ultra-long protocol is associated with severe endometriosis which was an exclusion criteria for the trial.
Exclusion criteria added for participants planned to undergo a scratch or similar procedure (e.g. endometrial biopsy for the collection of natural killer cells).	Added given there are safety concerns around a participant receiving more than one endometrial scratch type procedure in a short space of time.
Clarification that a participant should still remain in the trial (and follow-up carried out) if the intervention is not carried out.	Following misunderstandings at the research sites that participants should be withdrawn from the trial if the intervention isn't carried out.
Detail added regarding collection of safety events at the time of pregnancy tests (sites should attempt to collect safety events from notes if the participant is not contactable following a negative pregnancy test).	Research sites found it difficult to contact participants following a negative pregnancy test. Therefore, we recommended to sites that they reviewed the medical notes to identify any adverse events.
Conditions added to list of expected adverse events (anaemia, cholecystitis, epistaxis, itchy skin).	Identified following a review of reported adverse events. These events were commonly reported, and, in order to reduce the<br>Burden of reporting such events, were added to the list of expected adverse events.
Electronic questionnaires added at 3 month and 6 week post partum follow-up, in addition to the already approved paper versions of the questionnaires at the same time points.	Added in order to improve response rates to the questionnaire.
Summary letter introduced to be sent to participants who achieve a pregnancy in order to inform when the 3 telephone follow-up s will be undertaken.	Added in order to improve the response rate to the 3 telephone follow-ups.
**Version 5 (20/07/2017) amended to version 6 (13/11/2017) [Dundee only]**	
A separate protocol was created for the Dundee research site, where a postal consent process was implemented. Participants were approached at their routine visit to the clinic, and if interested, were asked to sign and return a postal consent form to the unit stating they were happy to be randomised into the trial. Randomisation then took place, and, if randomised to do so, the participant was scheduled an endometrial scratch procedure.	Added due to issues with participants having to travel vast distances to undertake consent and randomisation activities
**Version 6 (13/11/2017) [Dundee only] and Version 5 (20/07/2017) [all other sites], amended to version 7 (28/01/2019) [Dundee only] and version 6 (28/01/2019) [all other sites]**	
The number of centres participating in the trial was updated—this was stated in previous versions of the protocol as "approximately 10", but was updated to the final number of sites (n = 16).	Updated following an increase in the number of sites participating in the trial.
Clarification was provided regarding the follow-up of trial participants who undergo frozen embryo transfer (FET), where the first FET (where no previous transfer has been undertaken) will be followed up within the trial.	In order to improve clarity.
Additional information was added regarding the randomisation process, including allocation concealment.	Added in order to ensure compliance with CONSORT guidelines.
The qualitative substudy was added and systematic review.	Extra components added to the trial following approval of these elements by the funder.
Changes were made to the reporting of adverse events—events related to the birth of a baby or the process of birth were no longer to be classed as adverse events or collected within the trial, events related to the weight or growth of the baby (low birth weight, very low birth weight, large for gestational age, preterm delivery, very preterm delivery and small for gestational age) were no longer to be classed as adverse events, but would still be reported, events were added to the list of expected adverse events in pregnancy [back pain, sciatica, bloating, breast tenderness/pain, cold/flu, feeling faint, fatigue/tiredness, frequent urination/nocturia, migraine, hot flushes, hyperemesis, implantation bleed, period pain, symphysis pubis dysfunction/hip pain/pelvis dysplasia, placenta previa (grade 1 and 2), prolapse, reflux/heartburn/indigestion[and during IVF treatment (abdominal swelling associated with mild OHSS, mild OHSS). Possible foetal abnormality was removed as an expected adverse event.	The reporting of these events was causing a significant burden to research sites. The trial steering committee approved the declassification of these events.
**Non-substantial amendments**	
The Primary Investigator at the South Tees trial site was changed from Ms. Mohar Goswami to Mr. Fayez Mustafa from February 2019, due to the relocation of Ms Goswami.	n/a
