# Supplementary material for: A randomised controlled trial to assess the clinical effectiveness and safety of the endometrial scratch procedure prior to first-time IVF, with or without ICSI
Source: Hum Reprod. 2021 May 29;36(7):1841–53. doi: 10.1093/humrep/deab041 (PMC8213451; doi:10.1093/humrep/deab041)
Supplement: deab041_Supplementary_Table_S3 [file deab041_supplementary_table_s3.pdf]

Human Reproduction, pp. 1–1, 2021
doi:10.1093/humrep/deab041

human reproduction

SUPPLEMENTARY DATA

Supplementary Table SIII **Blastocyst embryo grading.**

Category	Gardners	New NEQAS	Old NEQAS
Excellent	A/A	A/A	5/3
Very good	A/B, B/A	A/B, B/A	5/2, 4/3, 3/4, 4/4
Good	B/B	B/B	4/2,
Fair + freezable	A/C, C/A	BC/CB, A/C	3/3, 3/2
Fair	B/C, C/B	C/C, A/D	3/1, 4/1, 5/1<br>2/3, 2/2, 1/3
Poor	C/C, Degree of expansion 2 and X/X	C/D, D/C, D/D, Degree of expansion 2 and X/X	2/1, 1/1, 1/2
Early blastocyst	No grades provided (X/X/X)<br>Degree of expansion 1 and any other TE or ICM grade	No grades provided (X/X/X)<br>Degree of expansion 1 and any other TE or ICM grade	No grades provided (X/X/X)<br>Degree of expansion 1 and any other TE or ICM grade

ICM, inner cell mass; NEQAS, National External Quality Assessment Service; TE, trophectoderm; X, no grade provided.
Old NEQAS: cell number/shape score/fragmentation score.
New NEQAS: cell number/blastomere size/fragmentation score.
