# Supplementary material for: A randomised controlled trial to assess the clinical effectiveness and safety of the endometrial scratch procedure prior to first-time IVF, with or without ICSI
Source: Hum Reprod. 2021 May 29;36(7):1841–53. doi: 10.1093/humrep/deab041 (PMC8213451; doi:10.1093/humrep/deab041)
Supplement: deab041_Supplementary_Table_S4 [file deab041_supplementary_table_s4.pdf]

Human Reproduction, pp. 1–1, 2021
doi:10.1093/humrep/deab041

human reproduction

SUPPLEMENTARY DATA

Supplementary Table SIV **Cleavage stage embryo grading.**

Category	NEQAS (pre/post April 2017) grading on day 2 of development	NEQAS (pre/post April 2017) grading on day 3 of development	NEQAS (pre/post April 2017) grading on day 5 of development
**Excellent**	4/4/4, 3/4/4	8/4/4	N/A
**Good**	5/4/4, 5/3/4, 5/4/3, 5/3/3, 4/3/4, 4/4/3, 4/3/3	10/4/4, 10/4/3, 10/3/4, 10/3/3, 9/4/4, 9/4/3, 9/3/4, 9/3/3, 8/4/3, 8/3/4, 8/3/3, 7/4/4, 7/4/3, 7/3/3, 6/4/4, 6/3/4, 6/4/3, 6/3/3, 7/3/4	N/A
**Fair**	5/2/3, 5/2/4, 4/2/3, 3/3/4, 3/3/3, 3/2/3, 4/2/4	6/2/4, 8/4/2	N/A
**Poor quality**	all >6c combinations, 5/3/2, 5/2/2, 4/3/2, 4/2/2, 3/4/3, 3/3/2, 3/2/2,	all >11c combinations, 10/3/2, 10/2/3, 10/2/2, 9/3/2, 9/2/3, 9/2/2, 8/3/2, 8/2/3, 8/2/2, 7/3/2, 7/2/3, 7/2/2, 6/3/2, 6/2/30, 6/2/2	N/A
**Very poor quality**	5/2/1, 5/1/2, 5/1/1, 4/2/1, 4/1/2, 4/1/1, 3/2/1, 3/1/2, 3/1/1, 3/1/3	all -/1/1 combinations, 5/2/1	N/A
**Slow**	All 2c combinations	All $\leq$ 5 cell combinations except -/1/1, 5/4/4, 5/3/4, 5/4/3, 5/3/3, 5/2/3, 5/3/2, 5/2/2, 5/2/3	N/A
**Arrested development**	N/A	N/A	Any cleavage grading

-, any cell number; C, cell; D, day; N/A, not applicable.
Old NEQAS: cell number/shape score/fragmentation score.
New NEQAS: cell number/blastomere size/fragmentation score.
