# Supplementary material for: A randomised controlled trial to assess the clinical effectiveness and safety of the endometrial scratch procedure prior to first-time IVF, with or without ICSI
Source: Hum Reprod. 2021 May 29;36(7):1841–53. doi: 10.1093/humrep/deab041 (PMC8213451; doi:10.1093/humrep/deab041)
Supplement: deab041_Supplementary_Table_S5 [file deab041_supplementary_table_s5.pdf]

Human Reproduction, pp. 1–1, 2021
doi:10.1093/humrep/deab041

human reproduction

SUPPLEMENTARY DATA

Supplementary Table SV **Summary of self-reported pain rating of endometrial scratch procedure.**

Timing post ES procedure	Summary	Pain rating summary	
		Including ambiguous scores	Excluding ambiguous scores
Within 30 minutes		(n = 450)	(n = 450)
	Mean (SD)	4.1 (2.4)	4.1 (2.4)
	Median (IQR)	4.0 (2.0, 6.0)	4.0 (2.0, 6.0)
	Min, max	0.0, 10.0	0.0, 10.0
Day 1		(n = 408)	(n = 402)
	Mean (SD)	1.8 (2.5)	1.8 (2.5)
	Median (IQR)	1.0 (0.0, 3.0)	1.0 (0.0, 3.0)
	Min, max	0.0, 10.0	0.0, 10.0
Day 7		(n = 397)	(n = 390)
	Mean (SD)	0.8 (2.1)	0.8 (2.0)
	Median (IQR)	0.0 (0.0, 0.0)	0.0 (0.0, 0.0)
	Min, max	0.0, 10.0	0.0, 10.0

The higher the score the greater the pain: 0 (no pain) to 10 (worst pain imaginable); ES: endometrial scratch, IQR, interquartile range; min, minimum; max, maximum.
