# Supplementary material for: A randomised controlled trial to assess the clinical effectiveness and safety of the endometrial scratch procedure prior to first-time IVF, with or without ICSI
Source: Hum Reprod. 2021 May 29;36(7):1841–53. doi: 10.1093/humrep/deab041 (PMC8213451; doi:10.1093/humrep/deab041)
Supplement: deab041_Supplementary_Table_S6 [file deab041_supplementary_table_s6.pdf]

Human Reproduction, pp. 1–1, 2021
doi:10.1093/humrep/deab041

human reproduction

SUPPLEMENTARY DATA

Supplementary Table SVI Treatment cycle characteristics before egg collection.

Characteristic	TAU (n = 494)	ES (n = 497)	Total (n = 991)
Treatment protocol followed, n(%)			
Antagonist	297 (60.1%)	291 (58.6%)	588 (59.3%)
Long protocol	197 (39.9%)	205 (41.2%)	402 (40.6%)
Ultra-long protocol ‡	0 (0.0%)	1 (0.2%)	1 (0.1%)
FSH drug used, n(%)			
Gonal F	153 (29.1%)	158 (30.2%)	311 (29.7%)
Merional	127 (24.2%)	118 (22.6%)	245 (23.4%)
Menopur	127 (24.2%)	135 (25.8%)	262 (25.0%)
Bemfola	46 (8.8%)	46 (8.8%)	92 (8.8%)
Fostimon	8 (1.5%)	7 (1.3%)	15 (1.4%)
Meriofert	27 (5.1%)	24 (4.6%)	51 (4.9%)
Ovaleap	4 (0.8%)	4 (0.8%)	8 (0.8%)
Rekovelle	1 (0.2%)	1 (0.2%)	2 (0.2%)
Bemfola and Menopur	1 (0.2%)	0 (0.0%)	1 (0.1%)
Gonal F and Menopur	0 (0.0%)	2 (0.4%)	2 (0.2%)
Merional and Fostimon	0 (0.0%)	1 (0.2%)	1 (0.1%)
Class of FSH drug used, n(%)			
Recombinant	204 (38.9%)	209 (40.0%)	413 (39.4%)
Purified urinary	289 (55.0%)	285 (54.5%)	574 (54.8%)
Purified urinary and recombinant combination	1 (0.2%)	2 (0.4%)	3 (0.3%)
Purified urinary combination	0 (0.0%)	1 (0.2%)	1 (0.1%)
Planned treatment protocol changed, n(%)			
No	488 (98.8%)	489 (98.4%)	977 (98.6%)
Yes	6 (1.2%)	8 (1.6%)	14 (1.4%)
Trigger, n(%)			
hCG	468 (94.7%)	456 (91.8%)	924 (93.2%)
Agonist	18 (3.6%)	28 (5.6%)	46 (4.6%)
Not administered	8 (1.6%)	13 (2.6%)	21 (2.1%)
Number of days of FSH (days)	(n = 494)	(n = 496)	(n = 990)
Mean(SD)	10.9 (1.9)	11.0 (2.2)	10.9 (2.0)
Min, max	5.0, 19.0	3.0, 30.0	3.0, 30.0
Performed cycle programming, n(%)	124 (23.6%)	118 (22.6%)	242 (23.1%)
Details of cycle programming	(n = 124)	(n = 118)	(n = 242)
Oral contraception, n(%)	64 (51.6%)	63 (53.4%)	127 (52.5%)
Progestogens, n(%)	53 (42.7%)	48 (40.7%)	101 (41.7%)
Oral oestrogen, n(%)	7 (5.6%)	7 (5.9%)	14 (5.8%)
Taking any medications that contraindicate IVF/ICSI, n(%)	0 (0.0%)	0 (0.0%)	0 (0.0%)

‡one participant was randomised prior to ultra-long protocols being excluded from the trial. This participant was stratified as long-protocol during randomisation.
TAU, treatment as usual; Recombinant: Gonal F, Bemfola, Rekovelle, and Ovaleap; Purified urinary: Merional, Menopur, Fostimon, and Meriofert; Purified urinary and recombinant combination: Bemfola and Menopur, Gonal F and Menopur; Purified urinary FSH combination: Merional and Fostimon. = min, minimum; max, maximum.
