# Supplementary material for: A randomised controlled trial to assess the clinical effectiveness and safety of the endometrial scratch procedure prior to first-time IVF, with or without ICSI
Source: Hum Reprod. 2021 May 29;36(7):1841–53. doi: 10.1093/humrep/deab041 (PMC8213451; doi:10.1093/humrep/deab041)
Supplement: deab041_Supplementary_Table_S7 [file deab041_supplementary_table_s7.pdf]

Human Reproduction, pp. 1–1, 2021
doi:10.1093/humrep/deab041

human reproduction

SUPPLEMENTARY DATA

Supplementary Table SVII **Treatment cycle characteristic at egg collection.**

Characteristic	TAU (n = 494)	ES (n = 497)	Total (n = 991)
Eggs collected, n(%)	482 (97.6%)	481 (96.8%)	963 (97.2%)
Eggs not collected with reasons, n(%)	12 (2.4%)	16 (3.2%)	28 (2.8%)
Empty follicles, n	1	1	2
Poor response, n	8	12	20
Participant withdrew from IVF/trial, n	1	1	2
Other ‡, n	2	2	4
Number of eggs collected	(n = 482)	(n = 481)	(n = 963)
Mean(SD)	12.0 (6.4)	12.0 (6.6)	12.0 (6.5)
Median(IQR)	11.0 (8.0, 15.0)	11.0 (8.0, 16.0)	11.0 (8.0, 15.0)
Min, max	1.0, 39.0	1.0, 44.0	1.0, 44.0
	(n = 482)	(n = 481)	(n = 963)
Successful egg fertilisation, n(%)	476 (98.8%)	465 (96.7%)	941 (97.7%)
Method of fertilisation			
IVF	264 (55.5%)	241 (51.8%)	505 (53.7%)
ICSI	205 (43.1%)	209 (44.9%)	414 (44.0%)
IVF and ICSI split	7 (1.5%)	15 (3.2%)	22 (2.3%)
Sperm used, n(%)			
Fresh	434 (91.2%)	421 (90.5%)	855 (90.9%)
Frozen	42 (8.8%)	44 (9.5%)	86 (9.1%)
Type of sperm used, n(%)			
Ejaculate	432 (90.8%)	429 (92.3%)	861 (91.5%)
Donor	24 (5.0%)	20 (4.3%)	44 (4.7%)
PESA	6 (1.3%)	8 (1.7%)	14 (1.5%)
TESA	2 (0.4%)	3 (0.6%)	5 (0.5%)
TESE	10 (2.1%)	3 (0.6%)	13 (1.4%)
MicroTESE	2 (0.4%)	2 (0.4%)	4 (0.4%)
Method of fertilisation changed since plan, n(%)	26 (5.5%)	28 (6.0%)	54 (5.7%)
Sperm quality, n	23	23	46
Other ‡, n	3	5	8
Embryos generated day 1 after fertilisation, n(%)	475 (99.8%)	464 (99.8%)	939 (99.8%)
Number of embryos generated	(n = 475)	(n = 464)	(n = 939)
Mean(SD)	7.0 (4.6)	7.0 (4.5)	7.0 (4.5)
Median(IQR)	6.0 (4.0, 9.0)	6.0 (4.0, 9.0)	6.0 (4.0, 9.0)
Min, max	1.0, 28.0	1.0, 31.0	1.0, 31.0

PESA, percutaneous epididymal sperm aspiration; TESA, testicular sperm aspiration; TESE, testicular sperm extraction; MicroTESE, microsurgical testicular sperm extraction. ‡ other reasons for not collecting eggs (n = 4): cycle cancelled due to no maturing follicles (n = 1), cycle abandoned after 11 days as tachycardia and thyroid functional tests abnormal and high risk with general anaesthetic (n = 1), cycle cancelled as the participant has ovarian hyperstimulation syndrome (OHSS) (n = 1), and MicroTESE proved unsuccessful as no sperm were retrieved (n = 1); ‡other reasons for the change in fertilisation method (n = 8): low sperm concentration (n = 1), no sperm and donor back up used (n = 1), the patient suffered a bereavement so plan was changed to create an embryo and freeze them (n = 1), prolonged ovarian stimulation resulted in the cancellation of planned surgical sperm retrieval and treatment converted to IVF using donor sperm (n = 1), the patient could not produce semen and semen was produced at home so there was a time delay with sperm production (n = 1), poor response to sperm preparation (n = 1), patient choice (n = 1), and hyperstimulation (n = 1).
