# Supplementary material for: A randomised controlled trial to assess the clinical effectiveness and safety of the endometrial scratch procedure prior to first-time IVF, with or without ICSI
Source: Hum Reprod. 2021 May 29;36(7):1841–53. doi: 10.1093/humrep/deab041 (PMC8213451; doi:10.1093/humrep/deab041)
Supplement: deab041_Supplementary_Table_S8 [file deab041_supplementary_table_s8.pdf]

Human Reproduction, pp. 1–2, 2021
doi:10.1093/humrep/deab041

human reproduction

SUPPLEMENTARY DATA

Supplementary Table SVIII **Treatment cycle characteristics at embryo transfer.**

Characteristic‡	TAU (n = 475)	ES (n = 464)	Total (n = 939)
Embryos transferred, n(%)	464 (97.7%)	455 (98.1%)	919 (97.9%)
Fresh, n(%)	425 (91.6%)	416 (91.4%)	841 (91.5%)
Frozen, n(%)	39 (8.4%)	39 (8.6%)	78 (8.5%)
Reasons for not transferring fresh embryos, n(%)	(n = 50)	(n = 48)	(n = 98)
Abnormal uterine cavity	2 (4.0%)	3 (6.3%)	5 (5.1%)
Hyperstimulation	32 (64.0%)	30 (62.5%)	62 (63.3%)
No suitable embryos to transfer	7 (14.0%)	6 (12.5%)	13 (13.3%)
Other ‡	9 (18.0%)	9 (18.8%)	18 (18.4%)
Difficult embryo transfer, n(%)	24 (5.2%)	28 (6.2%)	52 (5.7%)
Number of embryos transferred, n(%)			
Single	374 (80.6%)	381 (83.7%)	755 (82.2%)
Double	90 (19.4%)	74 (16.3%)	164 (17.8%)
Day of embryo transfer, n(%)	(n = 464)	(n = 455)	(n = 919)
2	27 (5.8%)	29 (6.4%)	56 (6.1%)
3	57 (12.3%)	68 (14.9%)	125 (13.6%)
4	11 (2.4%)	6 (1.3%)	17 (1.8%)
5	367 (79.1%)	352 (77.4%)	719 (78.2%)
6	2 (0.4%)	0 (0.0%)	2 (0.2%)
Type of catheter used, n(%)	(n = 464)	(n = 455)	(n = 919)
COOK K Jet	81 (17.5%)	90 (19.8%)	171 (18.6%)
COOK Guardia Access	137 (29.5%)	122 (26.8%)	259 (28.2%)
Wallace Classic/1816	79 (17.0%)	70 (15.4%)	149 (16.2%)
Wallace Sure-Pro	120 (25.9%)	131 (28.8%)	251 (27.3%)
Labotect	2 (0.4%)	1 (0.2%)	3 (0.3%)
Rocket Embryon	21 (4.5%)	19 (4.2%)	40 (4.4%)
Wallace SureView	24 (5.2%)	22 (4.8%)	46 (5.0%)
Blastocyst 1, n (%); quality	355 (76.5%)	345 (75.8%)	700 (76.2%)
	(n = 464)	(n = 455)	(n = 919)
Excellent	87 (18.8%)	102 (22.4%)	189 (20.6%)
Very good	92 (19.8%)	109 (24.0%)	201 (21.9%)
Good	95 (20.5%)	74 (16.3%)	169 (18.4%)
Fair and freezable	35 (7.5%)	28 (6.2%)	63 (6.9%)
Fair	21 (4.5%)	15 (3.3%)	36 (3.9%)
Poor	13 (2.8%)	7 (1.5%)	20 (2.2%)
Early blastocyst	12 (2.6%)	10 (2.2%)	22 (2.4%)
Blastocyst 2, n (%); quality	30 (6.5%)	22 (4.8%)	52 (5.7%)
	(n = 464)	(n = 455)	(n = 919)
Excellent	1 (0.2%)	0 (0.0%)	1 (0.1%)
Very good	2 (0.4%)	3 (0.7%)	5 (0.5%)
Good	5 (1.1%)	2 (0.4%)	7 (0.8%)
Fair and freezable	9 (1.9%)	3 (0.7%)	12 (1.3%)
Fair	4 (0.9%)	3 (0.7%)	7 (0.8%)

(continued)

Supplementary Table SVIII Continued

Characteristic[‡]	TAU (n = 475)	ES (n = 464)	Total (n = 939)
Poor	5 (1.1%)	3 (0.7%)	8 (0.9%)
Early blastocyst	4 (0.9%)	8 (1.8%)	12 (1.3%)
Cleavage 1, n (%); quality	77 (16.6%)	94 (20.7%)	171 (18.6%)
	(n = 464)	(n = 455)	(n = 919)
Excellent	5 (1.1%)	7 (1.5%)	12 (1.3%)
Good	34 (7.3%)	41 (9.0%)	75 (8.2%)
Fair	2 (0.4%)	4 (0.9%)	6 (0.7%)
Poor	15 (3.2%)	15 (3.3%)	30 (3.3%)
Very poor	2 (0.4%)	1 (0.2%)	3 (0.3%)
Slow	7 (1.5%)	10 (2.2%)	17 (1.8%)
Arrested development	1 (0.2%)	0 (0.0%)	1 (0.1%)
Ungraded ‡‡	11 (2.4%)	16 (3.5%)	27 (2.9%)
Cleavage 2, n (%); quality	43 (9.3%)	40 (8.8%)	83 (9.0%)
	(n = 464)	(n = 455)	(n = 919)
Excellent	4 (0.9%)	1 (0.2%)	5 (0.5%)
Good	9 (1.9%)	10 (2.2%)	19 (2.1%)
Fair	1 (0.2%)	2 (0.4%)	3 (0.3%)
Poor	16 (3.4%)	12 (2.6%)	28 (3.0%)
Slow	5 (1.1%)	10 (2.2%)	15 (1.6%)
Arrested development	1 (0.2%)	0 (0.0%)	1 (0.1%)
Not graded (Gardners)	7 (1.5%)	5 (1.1%)	12 (1.3%)
Morula 1 (not graded), n(%)	32 (6.9%)	16 (3.5%)	48 (5.2%)
Morula 2 (not graded), n(%)	17 (3.7%)	12 (2.6%)	29 (3.2%)
Blood presence on tip of catheter, n(%)	34 (7.3%)	27 (5.9%)	61 (6.6%)
Fluid in the endometrium, n(%)	3 (0.6%)	4 (0.9%)	7 (0.8%)
Vulsellum used, n(%)	0 (0.0%)	5 (1.1%)	5 (0.5%)

[‡]The denominator is embryo generated;
[‡‡]grading was not done in sites that used the Gardners grading system. ‡ other reasons why fresh embryos were not transferred (n = 18): participant request to freeze embryos due to sudden family bereavement (n = 1); participant's request to freeze embryos (n = 1); reason for freeze is unknown (n = 1); personal issues (n = 1); unable to do the transfer (n = 1); acutely anteverted anteflexed uterus, scarring, adhesions at endocervix (n = 1); hydrosalpinges requiring intervention (n = 3); risk of OHSS (n = 3); planned frozen embryo transfer which was a protocol violation (n = 1); raised progesterone (n = 3); endometrial polyp found after IVF cycle started (n = 2) and; breaking down of a relationship (n = 1).
