# Supplementary material for: A randomised controlled trial to assess the clinical effectiveness and safety of the endometrial scratch procedure prior to first-time IVF, with or without ICSI
Source: Hum Reprod. 2021 May 29;36(7):1841–53. doi: 10.1093/humrep/deab041 (PMC8213451; doi:10.1093/humrep/deab041)
Supplement: deab041_Supplementary_Table_S9 [file deab041_supplementary_table_s9.pdf]

Human Reproduction, pp. 1–1, 2021
doi:10.1093/humrep/deab041

human reproduction

SUPPLEMENTARY DATA

Supplementary Table SIX **Effect of ES on live birth (primary outcome) based on per protocol analysis population.**

Live birth rate ‡	TAU	ES	Unadjusted treatment effect (95% CI)			P-value
	n/N (%)	n/N (%)	Absolute difference	Odds ratio	Relative risk	
PP, worst-case	169/417 (40.5%)	164/372 (44.1%)	3.6% (−3.3%, 10.5%)	1.16 (0.87, 1.54)	1.09 (0.92 , 1.28)	0.312
PP, best-case	170/417 (40.8%)	164/372 (44.1%)	3.3% (−3.6%, 10.2%)	1.15 (0.86, 1.52)	1.08 (0.92 , 1.27)	0.346
PP, complete case	169/416 (40.6%)	164/372 (44.1%)	3.5% (−3.4%, 10.4%)	1.15 (0.87, 1.53)	1.09 (0.92 , 1.28)	0.326
Live birth rate ‡‡			Adjusted treatment effect (95% CI)			
PP, worst-case	169/417 (40.5%)	164/372 (44.1%)	3.4% (−3.4%, 10.2%)	1.16 (0.86, 1.54)	1.08 (0.92 , 1.27)	0.323
PP, best-case	170/417 (40.8%)	164/372 (44.1%)	3.2% (−3.6%, 10.0%)	1.14 (0.86, 1.53)	1.08 (0.92 , 1.27)	0.360
PP, complete case	169/416 (40.6%)	164/372 (44.1%)	3.3% (−3.5%, 10.1%)	1.15 (0.86, 1.54)	1.08 (0.92 , 1.27)	0.342

‡Unadjusted for any covariates except the intervention;
‡‡Adjusted for fixed stratification factors (site and planned treatment protocol) and potential prognostic factors (age, BMI, duration of infertility, history of pregnancy, and current smoking status); PP, per protocol; n, number of women who gave at least one live birth; N, the total number of women.
