# Supplementary material for: A randomised controlled trial to assess the clinical effectiveness and safety of the endometrial scratch procedure prior to first-time IVF, with or without ICSI
Source: Hum Reprod. 2021 May 29;36(7):1841–53. doi: 10.1093/humrep/deab041 (PMC8213451; doi:10.1093/humrep/deab041)
Supplement: deab041_Supplementary_Table_S10 [file deab041_supplementary_table_s10.pdf]

Human Reproduction, pp. 1–1, 2021
doi:10.1093/humrep/deab041

human reproduction

SUPPLEMENTARY DATA

Supplementary Table SX **Effects of ES on the secondary outcomes in the ITT population (worst-case).**

Secondary outcome	TAU (N = 525)	ES (N = 523)	Unadjusted treatment effect (95% CI)			P-value
			Absolute difference	Odds ratio	Relative risk	
Implantation rate	258 (49.1%)	253 (48.4%)	−0.8% (−6.8%, 5.3%)	0.97 (0.76, 1.24)	0.98 (0.87, 1.11)	0.804
Clinical pregnancy rate	213 (40.6%)	223 (42.6%)	2.1% (−3.9%, 8.0%)	1.09 (0.85, 1.39)	1.05 (0.91, 1.21)	0.497
Miscarriage rate	43 (8.2%)	32 (6.1%)	−2.1% (−5.2%, 1.0%)	0.73 (0.45, 1.17)	0.75 (0.48, 1.16)	0.193
Multiple birth rate	11 (2.1%)	6 (1.1%)	−0.9% (−2.5%, 0.6%)	0.54 (0.20, 1.48)	0.55 (0.20, 1.47)	0.224
Preterm delivery rate ‡	20 (3.8%)	14 (2.7%)	−1.1% (−3.3%, 1.0%)	0.69 (0.35, 1.39)	0.70 (0.36, 1.38)	0.301
Ectopic pregnancy rate	2 (0.4%)	1 (0.2%)	−0.2% (−0.8%, 0.5%)	0.50 (0.05, 5.54)	0.50 (0.05, 5.52)	0.565
Stillbirth rate	1 (0.2%)	1 (0.2%)	0.0% (−0.5%, 0.5%)	1.00 (0.06, 16.09)	1.00 (0.06, 16.01)	0.998

ITT: intention to treat, p-values not adjusted for multiple hypothesis tests.
Biochemical pregnancies were 20/525 (3.8%) in the TAU and 11/523 (2.1%) in the ES.
No pregnancies of unknown location reported.
‡2 born babies had missing gestational age so were assumed not to be preterm deliveries.
