# Supplementary material for: A randomised controlled trial to assess the clinical effectiveness and safety of the endometrial scratch procedure prior to first-time IVF, with or without ICSI
Source: Hum Reprod. 2021 May 29;36(7):1841–53. doi: 10.1093/humrep/deab041 (PMC8213451; doi:10.1093/humrep/deab041)
Supplement: deab041_Supplementary_Table_S11 [file deab041_supplementary_table_s11.pdf]

Human Reproduction, pp. 1–1, 2021
doi:10.1093/humrep/deab041

human reproduction

SUPPLEMENTARY DATA

Supplementary Table SXI Effects of ES on negative secondary outcomes in pregnant women.

Secondary outcome	TAU (N = 258)	ES (N = 253)	Unadjusted treatment effect (95% CI)			P-value
			Absolute difference	Odds ratio	Relative risk	
Miscarriage rate	43 (16.7%)	32 (12.6%)	−4.0% (−10.1%, 2.1%)	0.72 (0.44, 1.19)	0.76 (0.50, 1.16)	0.199
Multiple birth rate	11 (4.3%)	6 (2.4%)	−1.9% (−5.0%, 1.2%)	0.55 (0.20, 1.50)	0.56 (0.21, 1.48)	0.233
Preterm delivery rate ‡	20 (7.8%)	14 (5.5%)	−2.2% (−6.5%, 2.1%)	0.70 (0.34, 1.41)	0.71 (0.37, 1.38)	0.314
Ectopic pregnancy rate	2 (0.8%)	1 (0.4%)	−0.4% (−1.7%, 0.9%)	0.51 (0.05, 5.64)	0.51 (0.05, 5.59)	0.574
Stillbirth rate	1 (0.4%)	1 (0.4%)	0.0% (−1.1%, 1.1%)	1.02 (0.06, 16.39)	1.02 (0.06, 16.22)	0.989

p-values not adjusted for multiple hypothesis tests.
Biochemical pregnancies were 20/258 (7.8%) in the TAU and 11/253 (4.3%) in the ES.
No pregnancies of unknown location reported.
‡2 born babies had missing gestational age so were assumed not to be preterm deliveries.
