# Supplementary material for: A randomised controlled trial to assess the clinical effectiveness and safety of the endometrial scratch procedure prior to first-time IVF, with or without ICSI
Source: Hum Reprod. 2021 May 29;36(7):1841–53. doi: 10.1093/humrep/deab041 (PMC8213451; doi:10.1093/humrep/deab041)
Supplement: deab041_Supplementary_Table_S12 [file deab041_supplementary_table_s12.pdf]

Human Reproduction, pp. 1–1, 2021
doi:10.1093/humrep/deab041

human reproduction

SUPPLEMENTARY DATA

Supplementary Table SXII **Expected adverse events during the entire trial.**

**Adverse events**	**TAU (N = 537)**	**ES (N = 458)**
At least one expected AE	174 (32.4%)	144 (31.4%)
At least one expected AE category		
Abdominal pain	44 (8.2%)	37 (8.1%)
Anaemia	22 (4.1%)	14 (3.1%)
Back pain/sciatica	4 (0.7%)	5 (1.1%)
Bloating	8 (1.5%)	4 (0.9%)
Cholestasis	2 (0.4%)	4 (0.9%)
Clicky hip	1 (0.2%)	2 (0.4%)
Cold/flu	1 (0.2%)	1 (0.2%)
Conjunctivitis	1 (0.2%)	0 (0.0%)
Constipation	10 (1.9%)	10 (2.2%)
Cough	6 (1.1%)	2 (0.4%)
Diarrhoea	6 (1.1%)	2 (0.4%)
Dizziness/feeling faint	12 (2.2%)	8 (1.7%)
Epistaxis	4 (0.7%)	2 (0.4%)
Fall	8 (1.5%)	5 (1.1%)
Fatigue/tiredness	3 (0.6%)	5 (1.1%)
Gestational diabetes	7 (1.3%)	10 (2.2%)
Headache/migraine	35 (6.5%)	24 (5.2%)
Hyperemesis	1 (0.2%)	1 (0.2%)
Hypertension	4 (0.7%)	4 (0.9%)
Itchy skin	5 (0.9%)	3 (0.7%)
Nausea	73 (13.6%)	50 (10.9%)
Vomiting	39 (7.3%)	25 (5.5%)
Palpitations	13 (2.4%)	6 (1.3%)
Pelvic girdle pain or symphysis pubis dysfunction or hip pain/pelvis	11 (2.0%)	6 (1.3%)
Pre-eclampsia	4 (0.7%)	4 (0.9%)
Proteinuria	1 (0.2%)	1 (0.2%)
PV bleed	50 (9.3%)	33 (7.2%)
PV discharge	9 (1.7%)	0 (0.0%)
Rash	2 (0.4%)	2 (0.4%)
Reduced foetal movement	20 (3.7%)	13 (2.8%)
Reflux/heartburn/indigestion	2 (0.4%)	2 (0.4%)
Strep B infection	4 (0.7%)	1 (0.2%)
Urinary tract infection	6 (1.1%)	4 (0.9%)
Vaginal infection	5 (0.9%)	3 (0.7%)
Viral infection	3 (0.6%)	2 (0.4%)
Mild OHSS	19 (3.5%)	18 (3.9%)

AE, adverse event; PV, vaginal bleeding.
