# Supplementary material for: A randomised controlled trial to assess the clinical effectiveness and safety of the endometrial scratch procedure prior to first-time IVF, with or without ICSI
Source: Hum Reprod. 2021 May 29;36(7):1841–53. doi: 10.1093/humrep/deab041 (PMC8213451; doi:10.1093/humrep/deab041)
Supplement: deab041_Supplementary_Table_S13 [file deab041_supplementary_table_s13.pdf]

Human Reproduction, pp. 1–2, 2021
doi:10.1093/humrep/deab041

human reproduction

SUPPLEMENTARY DATA

Supplementary Table SXIII **Unexpected AEs and SAEs after the delivery of interventions.**

AE classification	TAU		ES		
	(N = 537)	n/TPy [IR]	(N = 458)	n/TPy [IR]	IRR (95% CI)
Unexpected AEs	91 (16.9%)	120/255.7 [0.47]	87 (19.0%)	114/248.6 [0.48]	1.02 (0.76, 1.38)
SAEs	50 (9.3%)	58/255.7 [0.22]	37 (8.1%)	49/248.6 [0.19]	0.88 (0.58, 1.34)
Unexpected SAEs	25 (4.7%)	27/255.7 [0.10]	22 (4.8%)	26/248.6 [0.10]	1.00 (0.56, 1.78)
Expected SAEs	28 (5.2%)	31/255.7 [0.12]	20 (4.4%)	23/248.6 [0.09]	0.77 (0.43, 1.37)
Unexpected AE category					
Pain	20 (3.7%)	21	13 (2.8%)	17	
OHSS	5 (0.9%)	5	7 (1.5%)	7	
GI-related issues	10 (1.9%)	12	10 (2.2%)	11	
Infection/inflammation	18 (3.4%)	18	21 (4.6%)	23	
Bleeding/blood related events	34 (6.3%)	38	21 (4.6%)	22	
Urinary-related issues	6 (1.1%)	6	2 (0.4%)	2	
Cardiac-related issues	6 (1.1%)	6	13 (2.8%)	14	
PV-related issues ‡	0 (0.0%)	0	1 (0.2%)	1	
Pregnancy specific issues	9 (1.7%)	10	8 (1.7%)	9	
Placenta-related issues	7 (1.3%)	8	4 (0.9%)	4	
Skin-related issues	5 (0.9%)	5	0 (0.0%)	0	
Events in baby/foetus	5 (0.9%)	5	4 (0.9%)	4	
Mental health	1 (0.2%)	2	1 (0.2%)	1	
Neuro-related issues	1 (0.2%)	1	1 (0.2%)	1	
Uterine abnormality	0 (0.0%)	0	4 (0.9%)	4	
Other	13 (2.4%)	14	21 (4.6%)	21	
Seriousness of SAE					
Life threatening	5 (0.9%)	5	2 (0.4%)	2	
Inpatient hospitalisation	36 (6.7%)	40	29 (6.3%)	37	
Prolongs hospitalisation	12 (2.2%)	12	8 (1.7%)	10	
Persistent or significant disability/incapacity	1 (0.2%)	1	0 (0.0%)	0	
Frequency of SAE					
Isolated	43 (8.0%)	48	34 (7.4%)	39	
Intermittent	4 (0.7%)	4	5 (1.1%)	8	
Continuous	2 (0.4%)	2	1 (0.2%)	1	
Missing ‡‡	0 (0.0%)	0	1 (0.2%)	1	
Unknown	4 (0.7%)	4	0 (0.0%)	0	
Intensity of SAE					
Mild	18 (3.4%)	18	7 (1.5%)	7	
Moderate	29 (5.4%)	32	23 (5.0%)	31	
Severe	8 (1.5%)	8	6 (1.3%)	10	
Missing	0 (0.0%)	0	1 (0.2%)	1	
Outcome of SAE					
Recovered	45 (8.4%)	50	32 (7.0%)	40	
Improved	7 (1.3%)	7	5 (1.1%)	7	
Unchanged	1 (0.2%)	1	1 (0.2%)	1	

(continued)

Supplementary Table SXIII Continued

AE classification	TAU		ES		
	**(N = 537)**	**n/TPy [IR]**	**(N = 458)**	**n/TPy [IR]**	**IRR (95% CI)**
Missing ‡‡	0 (0.0%)	0	1 (0.2%)	1	
Relation to ES					
Possible	n/a	n/a	1 (0.2%)	1	
Unlikely	n/a	n/a	10 (2.2%)	11	
Unrelated	n/a	n/a	25 (5.5%)	35	
Missing ‡‡	n/a	n/a	1 (0.2%)	1	
Not assessable	n/a	n/a	1 (0.2%)	1	

SAEs, serious adverse events; n/TPy [IR], number of all repeated events/total follow-up time in years [incidence rate per person-year]; IR, Incidence rate; IRR, incidence rate ratio; n/a, not applicable; GI, gastrointestinal; PV, vaginal bleeding.

‡did not occur within 24 hours of the ES procedure;

‡‡the frequency, intensity, outcome, and relationship to ES procedure of one unexpected SAE that resulted in inpatient hospitalisation could not be ascertained (SAE was identified from the final trial questionnaire and the site could not contact the participant to ascertain the details).
