# Supplementary material for: A randomised controlled trial to assess the clinical effectiveness and safety of the endometrial scratch procedure prior to first-time IVF, with or without ICSI
Source: Hum Reprod. 2021 May 29;36(7):1841–53. doi: 10.1093/humrep/deab041 (PMC8213451; doi:10.1093/humrep/deab041)
Supplement: deab041_Supplementary_Table_S14 [file deab041_supplementary_table_s14.pdf]

Human Reproduction, pp. 1–1, 2021
doi:10.1093/humrep/deab041

human reproduction

SUPPLEMENTARY DATA

Supplementary Table SXIV Unexpected AEs between ES and IVF (ES group only).

Adverse events classification	ES	
	(N = 458)	Total events
Unexpected AE	11 (2.4%)	13
SAE	1 (0.2%)	1
Unexpected SAE	1 (0.2%)	1
Expected SAE	0 (0.0%)	0
Unexpected AE category		
Pain	2 (0.4%)	2
Gastrointestinal related issues	2 (0.4%)	2
Infection/inflammation	1 (0.2%)	1
Cardiac-related issues	1 (0.2%)	1
Mental health	1 (0.2%)	1
Other	9 (2.0%)	9
Seriousness of SAE		
Inpatient hospitalisation	1 (0.2%)	1
Frequency of SAE		
Isolated	1 (0.2%)	1
Intensity of SAE		
Moderate	1 (0.2%)	1
Outcome of SAE		
Improved	1 (0.2%)	1
Relationship to ES		
Unlikely	1 (0.2%)	1
