# Supplementary material for: A randomised controlled trial to assess the clinical effectiveness and safety of the endometrial scratch procedure prior to first-time IVF, with or without ICSI
Source: Hum Reprod. 2021 May 29;36(7):1841–53. doi: 10.1093/humrep/deab041 (PMC8213451; doi:10.1093/humrep/deab041)
Supplement: deab041_Supplementary_Table_S15 [file deab041_supplementary_table_s15.pdf]

Human Reproduction, pp. 1–1, 2021
doi:10.1093/humrep/deab041

human reproduction

SUPPLEMENTARY DATA

Supplementary Table SXV SAE in babies born.

SAE classification	TAU (N = 270)	TAU Total events	ES (N = 226)	ES Total events	Absolute difference (95% CI)
Severe congenital abnormality	3 (1.1%)	3	0 (0.0%)	0	−1.1% (−2.4%, 0.1%)
Neonatal death	0 (0.0%)	0	0 (0.0%)	0	n/a
Other [a]	0 (0.0%)	0	4 (1.8%)	4	1.8% (0.1%, 3.5%)
Preterm delivery [b]	22 (8.1%)	30	12 (5.3%)	16	−2.8% (−7.2%, 1.5%)
Very preterm delivery [c]	1 (0.4%)	1	0 (0.0%)	0	−0.4% (−1.1%, 0.4%)
Low birthweight [d]	38 (14.1%)	44	13 (5.8%)	17	−8.3% (−13.5%, −3.2%)
Very low birthweight [e]	14 (5.2%)	17	3 (1.3%)	3	−3.9% (−6.9%, −0.8%)
Small for gestational age [f]	19 (7.0%)	19	6 (2.7%)	6	−4.4% (−8.1%, −0.7%)
Large for gestational age [g]	19 (7.0%)	19	13 (5.8%)	13	−1.3% (−5.6%, 3.0%)

[a]congenital brain abnormality at 12 weeks, lumbosacral myelomeningocele with an Arnold-Chiari malformation, hypoplastic left heart syndrome, and full pulmonary cardiac resuscitation; severe congenital abnormalities: congenital anomaly detected antenatally or postnatally. Common minor congenital anomalies as defined by the European Monitoring of Congenital Anomalies (EUROCAT) minor anomaly exclusion list were excluded as unexpected SAEs. These excluded anomalies are either minor (e.g. skin tags), or expected for the gestation (e.g. patent ductus arteriosus in babies born <37 weeks; neonatal death: death of a baby within 6 weeks of life; preterm delivery: delivery of a live birth of gestational age ≥24 and <37 weeks; very preterm delivery: delivery of a live birth of gestational age <24 weeks; low birthweight: delivery of a live birth with birthweight ≤ 2499 g or <10th centile for a given gestational age and baby sex; very low birthweight: delivery of a live birth with birthweight <1500 g or <5th centile for a given gestational age and baby sex; small for gestational age: delivery of a live birth with birthweight <10th centile for a given gestational age and baby sex; large for gestational age: delivery of a live birth with birthweight >95th centile for a given gestational age and baby sex.
