# Supplementary material for: A randomised controlled trial to assess the clinical effectiveness and safety of the endometrial scratch procedure prior to first-time IVF, with or without ICSI
Source: Hum Reprod. 2021 May 29;36(7):1841–53. doi: 10.1093/humrep/deab041 (PMC8213451; doi:10.1093/humrep/deab041)
Supplement: deab041_Supplementary_Figure_S1 [file deab041_supplementary_figure_s1.pdf]

Human Reproduction, pp. 1–1, 2021
doi:10.1093/humrep/deab041

human reproduction

SUPPLEMENTARY DATA

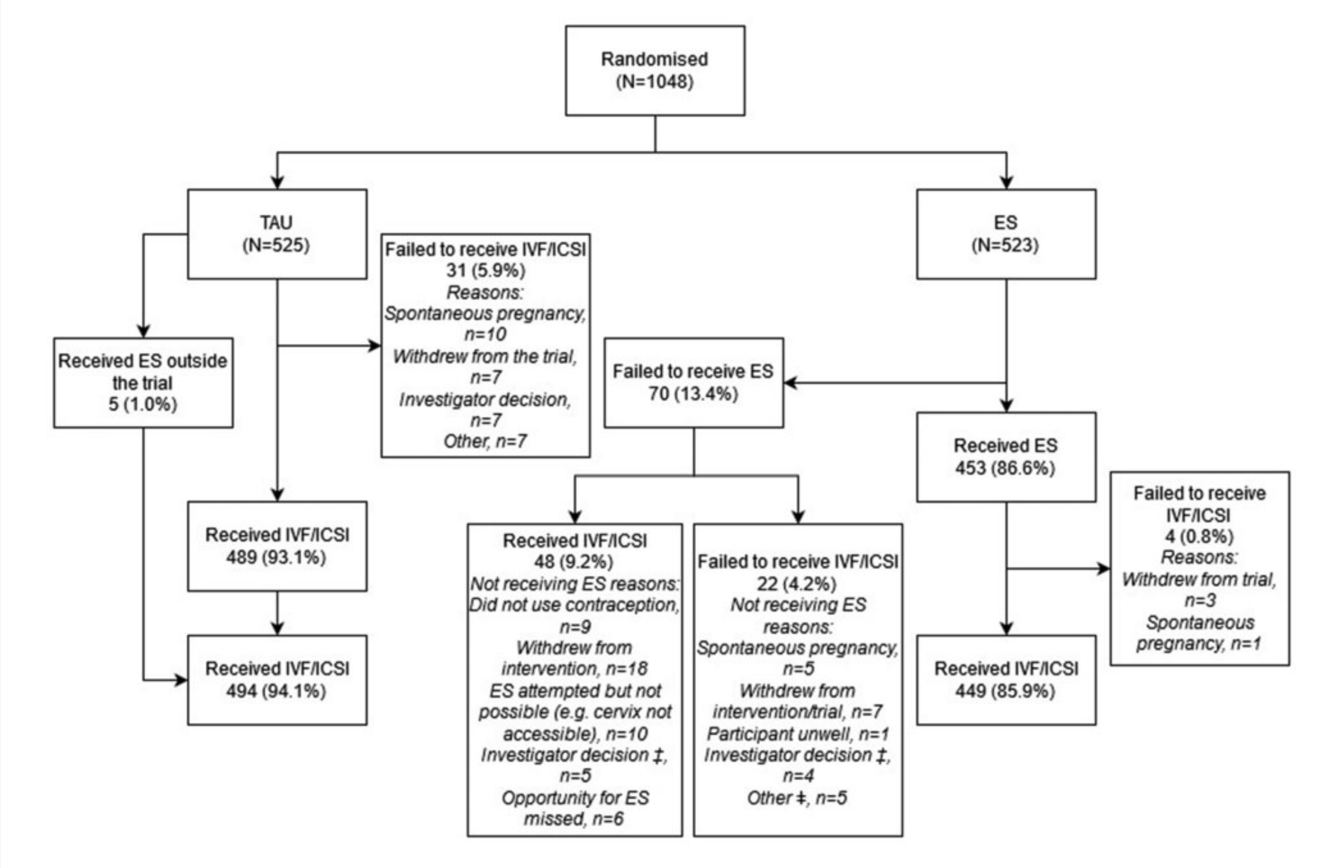

Supplementary Figure S1. Uptake of allocated treatments. ‡Other reasons (n = 5): lost to follow-up (n = 1), never returned to commence treatment (n = 3), and BMI was too high to start treatment (n = 1); ‡ investigator decisions (n = 9): deemed too risky to perform ES as the participant had a previous severe infection following IUI (n = 1); risk of perforation due to stenosis of the cervical canal (n = 1); lost to follow-up (n = 1); recently had surgery for adhesions (n = 1); had partial septate uterus on the scan (n = 1); identified adenomyosis within the uterus (n = 1); had a prostap (n = 2); participant had a scratch procedure in the past year which was noted when an endometrial biopsy was taken during exploratory surgery (n = 1).
