# Supplementary figures and images for: A randomised controlled trial to assess the clinical effectiveness and safety of the endometrial scratch procedure prior to first-time IVF, with or without ICSI
Source: Hum Reprod. 2021 May 29;36(7):1841–53. doi: 10.1093/humrep/deab041 (PMC8213451; doi:10.1093/humrep/deab041)

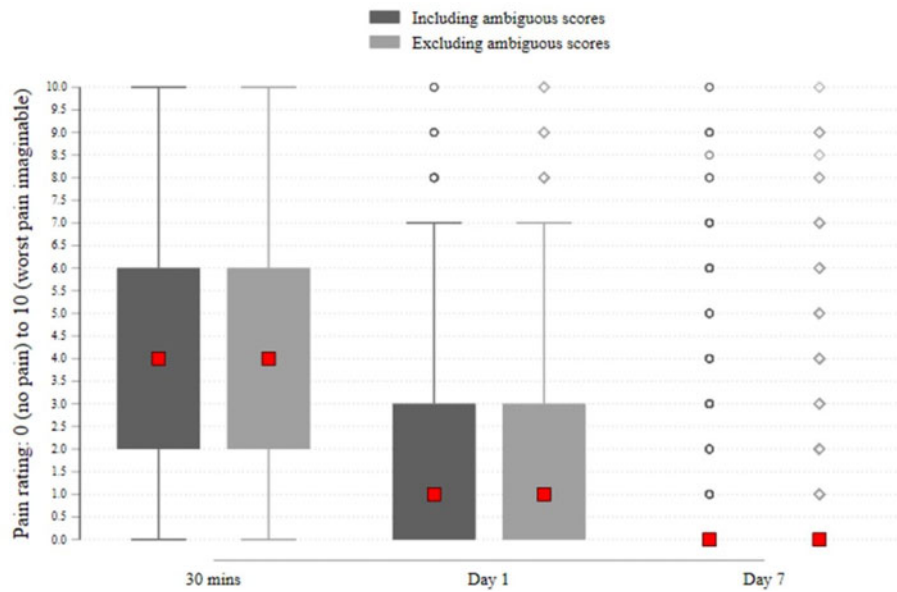

Supplementary Figure S3. Distribution of self-reported pain rating of ES procedure.

Supplement: deab041_Supplementary_Figure_S3 [file deab041_supplementary_figure_s3.pdf]
